# The Impact of Video-Based Microinterventions on Attitudes Toward Mental Health and Help Seeking in Youth: Web-Based Randomized Controlled Trial

**DOI:** 10.2196/54478

**Published:** 2024-04-24

**Authors:** Diana Lemmer, Markus Moessner, Nicolas Arnaud, Harald Baumeister, Agnes Mutter, Sarah-Lena Klemm, Elisa König, Paul Plener, Christine Rummel-Kluge, Rainer Thomasius, Michael Kaess, Stephanie Bauer

**Affiliations:** 1 Center for Psychotherapy Research Center for Psychosocial Medicine University Hospital Heidelberg Heidelberg Germany; 2 Ruprecht-Karls University Heidelberg Heidelberg Germany; 3 German Centre for Addiction Research in Childhood and Adolescence University Medical Centre Hamburg-Eppendorf Hamburg Germany; 4 Department of Clinical Psychology and Psychotherapy Ulm University Ulm Germany; 5 Department of Psychiatry and Psychotherapy University of Leipzig Medical Center Leipzig Germany; 6 Department of Child and Adolescent Psychiatry and Psychotherapy University Hospital Ulm Ulm Germany; 7 Department of Child and Adolescent Psychiatry Medical University of Vienna Vienna Austria; 8 University Hospital of Child and Adolescent Psychiatry and Psychotherapy University of Bern Bern Switzerland; 9 Clinic of Child and Adolescent Psychiatry Center for Psychosocial Medicine University Hospital Heidelberg Heidelberg Germany; 10 German Center for Mental Health (DZPG) Partner site Mannheim/Heidelberg/Ulm Heidelberg Germany

**Keywords:** help seeking, mental health, stigma, mental health literacy, psychoeducation, web-based experiment, web-based randomized controlled trial, microinterventions, video-based interventions

## Abstract

**Background:**

Mental health (MH) problems in youth are prevalent, burdening, and frequently persistent. Despite the existence of effective treatment, the uptake of professional help is low, particularly due to attitudinal barriers.

**Objective:**

This study evaluated the effectiveness and acceptability of 2 video-based microinterventions aimed at reducing barriers to MH treatment and increasing the likelihood of seeking professional help in young people.

**Methods:**

This study was entirely web based and open access. The interventions addressed 5 MH problems: generalized anxiety disorder, depression, bulimia, nonsuicidal self-injury, and problematic alcohol use. Intervention 1 aimed to destigmatize and improve MH literacy, whereas intervention 2 aimed to induce positive outcome expectancies regarding professional help seeking. Of the 2435 participants who commenced the study, a final sample of 1394 (57.25%) participants aged 14 to 29 years with complete data and sufficient durations of stay on the video pages were randomized in a fully automated manner to 1 of the 5 MH problems and 1 of 3 conditions (control, intervention 1, and intervention 2) in a permuted block design. After the presentation of a video vignette, no further videos were shown to the control group, whereas a second, short intervention video was presented to the intervention 1 and 2 groups. Intervention effects on self-reported potential professional help seeking (primary outcome), stigma, and attitudes toward help seeking were examined using analyses of covariance across and within the 5 MH problems. Furthermore, we assessed video acceptability.

**Results:**

No significant group effects on potential professional help seeking were found in the total sample (*F*_2,1385_=0.99; *P*=.37). However, the groups differed significantly with regard to stigma outcomes and the likelihood of seeking informal help (*F*_2,1385_=3.75; *P*=.02). Furthermore, separate analyses indicated substantial differences in intervention effects among the 5 MH problems.

**Conclusions:**

Interventions to promote help seeking for MH problems may require disorder-specific approaches. The study results can inform future research and public health campaigns addressing adolescents and young adults.

**Trial Registration:**

German Clinical Trials Register DRKS00023110; https://drks.de/search/de/trial/DRKS00023110

## Introduction

### Background

Mental health (MH) problems in youth are prevalent and pose severe health-related, social, and financial burdens on individuals [1-5] and societies [6,7]. Approximately half of all mental disorders first manifest before the age of 18 years [8], and MH problems in youth often persist and aggravate over the life span [9-14]. Therefore, the need for effective prevention and intervention programs targeting young people is an important public health goal. However, while effective MH services exist, most youth with MH problems do not seek professional help. Low uptake has been reported for various conventional [15-18] as well as digital MH services [19-22]. The burden of mental illness can only be alleviated at the population level if a substantial proportion of the population uses the available services [23,24]. Otherwise, the public health impact of MH services remains limited. Thus, increasing the reach of MH services (ie, fostering the uptake and use of professional help) is vital for the improvement of youth MH at the population level.

To facilitate service use, specific barriers to help seeking need to be addressed. Previous research has indicated that attitudinal factors pose larger impediments to help seeking than structural factors (eg, treatment costs and inconvenient scheduling) [25,26]. Specifically, self-reliance, a low perceived need for help [25-27], low treatment expectations [28], stigma [26,27,29,30], and poor MH literacy [27,31,32] have been identified as major contributors to the lack of professional help seeking.

Different approaches to facilitate help seeking and promote positive attitudes toward MH issues and help seeking in youth have been evaluated in previous research, including face-to-face and digital interventions. In a systematic mapping review, 84% (106/126) of the studies focused on school-based interventions, whereas only 10 (8%) articles covered internet-based approaches to improve MH literacy, MH-related attitudes, stigma, and help-seeking behavior in adolescents [33]. The internet-based interventions included both minimal, single-session interventions [34,35] and multisession approaches intended to be used over several weeks [36,37], with different outcome measures. A total of 4 studies focused on MH more broadly, whereas 6 studies investigated interventions for specific MH problems (depression: n=5; eating disorders: n=1). Keeping the limited number of studies in this area of research in mind, the results nevertheless point to the potential of internet-based interventions with respect to reduced stigma (2 studies), enhanced help-seeking intentions (2 studies), and improved help-seeking behaviors (1 study).

Clearly, there is a need for more research in this area, particularly with respect to digital brief and microinterventions (ie, highly focused in-the-moment interventions with a narrower scope and time frame than standard interventions [38]), which allow for a flexible, easily accessible, scalable, and efficient delivery of MH content. Initial research on such brief and microinterventions with psychoeducational and destigmatizing components has shown promising results. For instance, a brief acceptance-facilitating intervention that included a text-based personalized psychoeducation component had a small but significant effect on the intention to use MH services in German university students [39]. More recently, randomized controlled trials (RCTs) in young adults, university students, and adolescents with short video interventions demonstrated effects with regard to public stigma toward schizophrenia [40,41] and depression [42,43], as well as help-seeking intentions [42] and attitudes [43]. Furthermore, an Australian pilot study with international students found that a brief, web-based MH literacy intervention alleviated MH stigma. However, it had no significant effect on help-seeking intentions or MH literacy [44].

Another component of previous help seeking–facilitating strategies has been storytelling. A pilot study on a video-based intervention indicated that storytelling was well accepted and perceived as engaging [45]. In addition, an RCT evaluated internet-based storytelling programs with varying interactivity and stigma-related content. Significant reductions in MH stigma and microaggression toward individuals with MH problems were observed [46].

Concerning the theoretical foundation of interventions, few studies have investigated help seeking–promoting strategies that were explicitly based on the premises of health behavior models. Logsdon et al [47] evaluated an internet-based depression intervention for adolescent mothers, which was conceptualized according to the theory of planned behavior. The intervention led to significant improvements in help-seeking attitudes, intentions, and behavior. Another well-established and yet more recent health behavior model, which incorporates elements of previously developed approaches, is the Health Action Process Approach (HAPA) [48]. It encompasses a stage theoretical perspective on health behavior and includes a motivational, intention-forming phase as well as a volitional phase, where planning and behavior maintenance occur. In both the HAPA model and the updated version of the theory of planned behavior, namely, the reasoned action approach, outcome expectancies (or instrumental attitudes) play a crucial role in the formation of intentions, and intentions significantly predict actual behavior [49,50]. The results of previous research on a trauma recovery internet intervention support the use of the HAPA model for the prediction of e-MH engagement. Specifically, outcome expectations significantly predicted the intention to use the intervention (β=.36) [51]. Skepticism about treatment effectiveness has further been identified as a predictor for not using MH services in another study with university students [28].

Building on the findings of previous research, this study investigates the short-term effectiveness of 2 brief animated video interventions to promote potential professional help seeking in a general sample of adolescents and young adults aged 14 to 29 years using a web-based RCT approach. Both interventions aimed to improve participants’ willingness to seek professional help (ie, psychotherapists, psychiatrists, and counseling services) for 5 MH problems (generalized anxiety disorder [GAD], depression, bulimia, nonsuicidal self-injury [NSSI], and problematic alcohol use). The inclusion of various MH problems allowed for the investigation of potential differential effects. While one intervention followed a destigmatizing and psychoeducational approach, the other intervention aimed to induce positive outcome expectancies in accordance with the HAPA model through storytelling. The interventions were both compared to each other and to a nonintervention control group (CG) where participants were presented with a stand-alone video vignette without an additional intervention video. This approach was chosen due to both contextual (ie, vignette characters were described as experiencing difficulties in several life domains, and thus, additional control videos referring to the vignettes were unfeasible) and practical (ie, the creation of 10 additional videos was not necessary) considerations.

### Objectives

This study had the following objectives:

1. To investigate the short-term effectiveness of the 2 interventions in the promotion of potential MH help seeking (professional and informal), whereby self-reported professional help seeking was defined as the primary outcome.
2. To investigate the interventions’ effectiveness in the improvement of self-reported attitudes toward MH problems and MH service use (stigmatization and attitudes toward seeking MH services).
3. To evaluate the interventions’ self-reported acceptability.

Within the framework of this study, the videos were evaluated as stand-alone interventions. They were not developed to replace existing interventions. However, in case of favorable outcomes, they have the potential to complement existing health care services. Results and procedures are reported in accordance with the Checklist for Reporting Results of Internet E-Surveys [52] and the CONSORT-EHEALTH (Consolidated Standards of Reporting Trials of Electronic and Mobile Health Applications and Online Telehealth) [53]. The study was preregistered at the German Clinical Trials Register on September 23, 2020 (DRKS00023110).

## Methods

### Study Design

**Figure 1:**
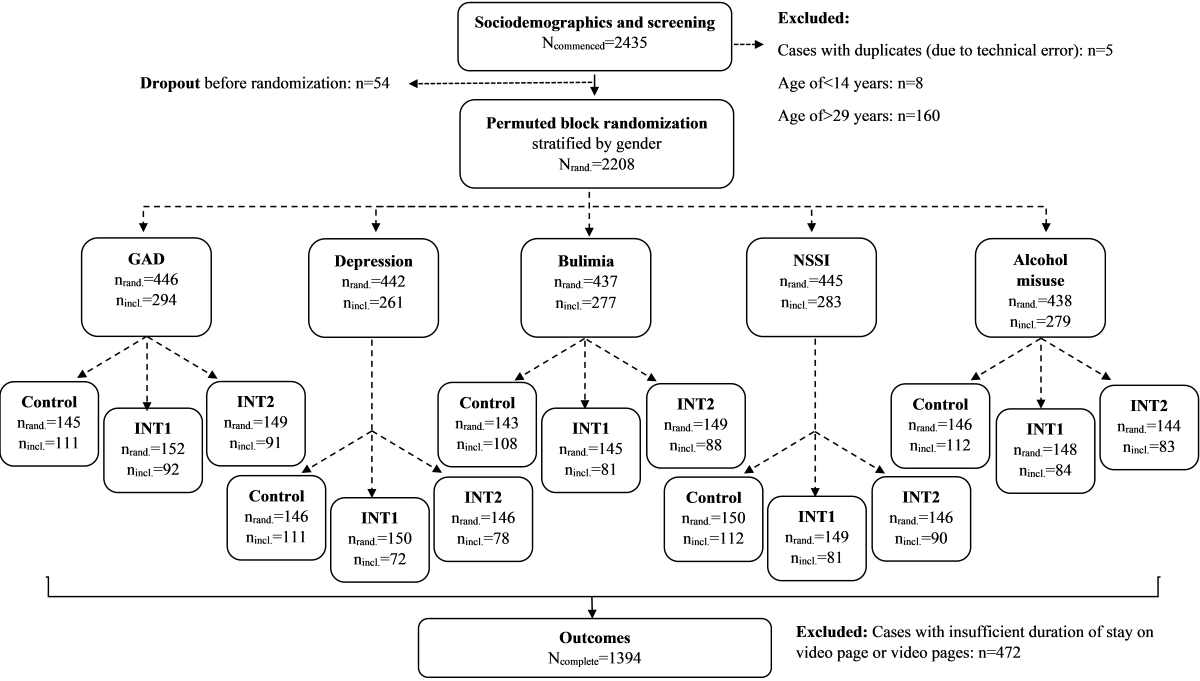
Study design and sample sizes. Participants were directly randomized into 1 of 15 experimental conditions. To enhance clarity and understanding, the figure illustrates the randomization process in 2 steps: mental health issue and experimental condition. GAD: generalized anxiety disorder; INT1: intervention 1; INT2: intervention 2; nincl: included cases (number of subgroup cases with complete data and sufficient duration on video page or video pages that were included in the data analysis); nrand: randomized cases (number of randomized subgroup cases irrespective of data completeness and video page duration); NSSI: nonsuicidal self-injury.

This anonymous, fully automated, web-based, parallel-group exploratory RCT compared the effects of intervention 1 (psychoeducational intervention) and intervention 2 (positive consequences of help seeking) against those of the CG (no further videos after the case vignette) with regard to potential help seeking, attitudes toward help seeking, and stigma. The design comprised 15 conditions in total (5 MH problems × 3 interventional conditions). Randomization was stratified by gender and implemented using a permuted block design (block sizes: 15 and 30). Due to anonymous participation and automated randomization, researchers were unable to assign specific conditions to individuals. However, 2 of the authors were able to view the randomization list. The video material was aligned with the participants’ gender to increase identification with the character (ie, participants who identified as woman, female, or nonbinary viewed videos with a female protagonist [Paula], and participants identifying as man or male viewed videos with a male protagonist [Paul]). The study components and conditions as well as the study procedure are shown in Figure 1.

### Recruitment and Sample

Recruitment started in October 2020 and ended in May 2022. Youth aged between 14 and 29 years with sufficient German language skills were eligible for participation. The age of 14 years is widely accepted as appropriate to provide informed consent for medical decisions and participation in studies [54,55]. The upper age limit of 29 years aligns with the definition of emerging adulthood, a separate life stage between adolescence and adulthood [56,57]. Participants were primarily recruited through the web on social media platforms and via mailing lists, web-based marketplaces, and forums for adolescents and young adults (eg, accounts and emails of youth clubs and student associations). As an incentive to complete the study, participants were offered to take part in an optional gift card lottery at the end of the study (100 gift cards of €20 [US $21.58]). We asked participants for a valid email address if they were interested in the lottery and stored email addresses separately from other study data and user IDs to ensure anonymous participation.

We recorded page change time stamps. Participants whose time stamp data indicated that the video or the videos they were assigned to had not been fully viewed (ie, duration of stay<length of the respective videos) were excluded from statistical analyses. Furthermore, only data from participants who completed all questionnaires were included in the final analysis (n=1394; completion rate: 1394/2435, 57.25%). We also excluded 5 cases with duplicate user IDs, which occurred due to a technical error and indicated repeated participation (Figure 1). HTTP cookies were used to assign individual user IDs to participants. For each session, new cookies were generated and used. Therefore, duplicate participation was possible after the completion of each study session and was not registered by the system. In the 5 aforementioned cases, duplicate IDs were mistakenly generated when participants tried to use the “back” button of their web browser and restarted their participation.

### Procedure

#### Overview

This study was conducted in an open access, voluntary web-based setting. A website was established to provide study information and enable participation. The ASMO software (Center for Psychotherapy Research) [58] was used to implement the RCT. A randomization list with numbers representing the conditions was generated and embedded in our ASMO database [58] before recruitment. Data were collected at the Center for Psychotherapy Research, Heidelberg. The study’s technical functionality and usability were extensively tested before recruitment by the authors and their colleagues at their respective institutions. Before their participation, the youth received detailed information about the aims, scope, procedures, data processing, and data storage of the study on the website. Participants were informed that they would be randomly assigned to 1 of 5 MH problems and 1 of 3 video versions. They were not informed about the specific health issues or the conditions’ details before participation. As the aim of the conditions was to provide information about a specific MH problem, blinding of participants after assignment to the interventions was not possible. Only participants who provided informed consent through a web-based checkbox were eligible for participation. After study completion, participants were debriefed in writing about the objectives on the study website. The debriefing form also included contact information for formal help services. Study duration amounted to approximately 30 minutes. Participants were first asked to complete sociodemographic and screening questionnaires; were then randomly assigned to 1 of the 15 experimental conditions; and, finally, were presented with the outcome questionnaires. The whole study (including informed consent and gift card lottery pages) comprised 26 pages with 1 to 12 items on each page. Each segment or measure was presented on 1 or 2 separate pages depending on its respective length. Some items were conditional for adaptive questioning (eg, when lifetime NSSI was denied, no further questions about NSSI were presented). Changes to the item responses could only be made while they had not been confirmed through a click on the “next” button, which brought participants to the next page. There was no “back” button.

#### Sociodemographics and Screening

All measures were self-reported. The sociodemographic form asked participants about their age, gender, migration background, education, whether they knew someone with MH problems, and participants’ previous or current MH service use (actual help seeking). Thereafter, participants’ subjective psychological distress was assessed using several screening instruments.

Anxiety symptoms were measured using the 7-item Generalized Anxiety Disorder Scale (GAD-7) [59]. Symptom frequency within the previous 2 weeks was indicated on a 4-point response scale. Total scores (potential range 0-21) were used for further analyses. Scores of ≥5 indicate a mild anxiety symptomatology, scores of ≥10 indicate a moderate anxiety symptomatology, and scores of ≥15 indicate a severe anxiety symptomatology [59].

The 9-item Patient Health Questionnaire (PHQ-9) [60] was used for depression symptomatology assessment. Frequencies of depression symptoms within the previous 2 weeks were measured on a 4-point scale. Total scores (potential range 0-27) were calculated for further analyses. Total scores of ≥5 were interpreted as mild, scores of ≥10 were interpreted as moderate, scores of ≥15 were interpreted as moderately severe, and scores of ≥20 were interpreted as severe depression symptomatology [60].

The Weight Concerns Scale (WCS) [61,62] assessed weight and body shape concerns. It consists of 5 items with varying response scale types (4- to 7-point scales). The response categories of each item represent scores between 0 and 100. The mean across all items was used for further analyses. Scores of ≥57 are indicative of a high risk of eating disorders [61].

Problematic alcohol use during the previous 12 months was measured using the Alcohol Use Disorders Identification Test for Consumption (AUDIT-C) [63,64]. It comprises 3 items with 5-point response scales. Sum scores range between 0 and 12. A score of 0 indicates abstinence, whereas scores between 1 and 3 are interpreted as moderate alcohol consumption. Scores of ≥4 indicate hazardous alcohol consumption [63,65].

A total of 4 items of the Self-Injurious Thoughts and Behaviors Interview [66] served to assess NSSI. The first item identified whether participants had ever harmed themselves without suicidal intention. If participants reported lifetime NSSI, the 3 subsequent questions were presented. These items measured the frequency of NSSI within the last year, the age at the first occurrence of NSSI, and the age at the last occurrence of NSSI. Item responses were analyzed separately and descriptively.

#### Experimental Conditions and Materials

##### Overview

The interventional strategies were applied using short animated videos. The videos were created with the Pro+ version of the web-based animation tool Powtoon (Powtoon Limited) [67]. Each research group involved in this study prepared materials for 1 of the 5 MH problems based on their respective field of expertise. The materials were structured in a similar fashion across MH problems. The main characters, Paul and Paula, were introduced as students aged 16 years in each condition. In total, 30 videos were created: 5 MH problems × 2 main character genders × 3 video types. Participants in the control condition only viewed a vignette, whereas participants in both intervention groups each viewed 1 additional video (either for intervention 1 or intervention 2). A subset of the videos was pretested between July 2020 and September 2020 with a convenience sample of 9 youths (mean age 18.56, SD 3.74 years; range 14-24 years; 3/9, 33% male), who confirmed comprehensibility and overall acceptability.

##### Vignettes

All participants viewed a case vignette. Each vignette depicted the respective main character, who was affected by 1 of 5 MH problems (GAD, depression, bulimia, NSSI, or problematic alcohol use). The vignettes introduced the characters to the viewers in a third-person perspective and described their challenges in their everyday lives due to their MH conditions (eg, difficult emotions and cognitions, physical symptoms, and social and school-related issues). The accurate diagnostic labels were not presented in the vignettes [68]. Vignette duration ranged from 2 minutes, 19 seconds to 2 minutes, 47 seconds (mean 2 min, 29 s; SD 11 s). The bulimia vignettes were developed first. They were inspired by the vignettes by Mond et al [69] and adapted in accordance with *International Classification of Diseases, 10th Revision* and *Diagnostic and Statistical Manual of Mental Disorders, 5th Edition* diagnostic criteria, as well as further literature on the symptomatology and psychological strain of bulimia [70]. The bulimia vignettes then served as a template for the vignettes of the other 4 MH problems.

##### Intervention 1

Intervention 1 aimed to improve MH literacy and decrease stigmatization through the presentation of psychoeducational information to encourage help seeking. These intervention videos first presented the correct diagnostic label, prevalence rates, and core symptoms of the condition shown in the vignette. Next, 5 destigmatizing and psychoeducational facts about the respective condition were presented (eg, “Bulimia is a serious illness and not a lifestyle”), which were inspired by the work by Bulik [71]. The videos then presented treatment options, information about potential challenges in professional help seeking, and encouraging statements about the benefits of professional MH support. Intervention 1 video durations ranged from 4 to 5 minutes (mean 4 min, 27 s; SD 21 s). The information provided in these intervention videos was based on epidemiological, etiological, diagnostic, barrier-related, and interventional findings on the respective MH problems (eg, the studies by Bulik [71], Keski-Rahkonen and Mustelin [72], and Nagl et al [73] for bulimia).

##### Intervention 2

The second strategy (intervention 2) was based on the premises of the HAPA [48]. Intervention 2 was designed to induce positive outcome expectancies of professional help seeking through the continuation of Paul and Paula’s stories. The videos showed the main characters 1 year after their initial situation as described in the vignettes. Intervention 2 videos first demonstrated the help-seeking process of the main characters in a retrospective fashion. Encouraged by their teachers, friends, or parents, the main characters sought and received professional support from a psychotherapist. The psychotherapist’s gender matched the gender of the main character. The videos showed how the psychotherapist informed the main character about the correct diagnostic label of their condition and shortly portrayed the therapeutic process. The process included initial difficulties of the main character, such as feelings of insecurity about disclosing their experiences to their therapist, which were resolved over time, and the main characters became invested in their psychotherapy. Then, 5 positive consequences of psychotherapy were presented, such as decreased impairment and an improved quality of life. The videos ended with the notion that the main character was still facing occasional difficulties, but substantial improvements in overall well-being and satisfaction with their decision to seek help were emphasized. Intervention 2 video durations ranged from 4 minutes, 1 second to 4 minutes, 29 seconds (mean 4 min, 15 s; SD 14 s). These interventions were designed in accordance with previous literature on the therapeutic process in MH conditions, including treatment expectations, experiences, and consequences [74].

#### Outcome Measures

##### Primary Outcome Measure

Our primary outcome was the potential use of professional MH services (ie, the hypothetical likelihood of seeking formal sources of help if participants experienced Paul’s or Paula’s MH problem), measured using a 12-item version of the General Help Seeking Questionnaire (GHSQ) [75]. The GHSQ measures the willingness of seeking various formal and informal sources of help within the next 4 weeks for an indicated MH problem on a 7-point rating scale (1=“extremely unlikely”; 7=“extremely likely”). The maximum score among the 3 items, which measured potential help seeking with professional MH services (psychotherapists, psychiatrists, and counseling services), was used as our primary outcome. The GHSQ is the most frequently used instrument for help seeking [76].

##### Secondary Outcome Measures

GHSQ data on the potential use of informal sources (romantic partner, friend, parent, or other family member) and no intended help seeking (1 item) were used as secondary outcomes. For informal sources of support, the items’ maximum score was used for the analyses.

Attitudes toward help seeking were measured using the Inventory of Attitudes Toward Seeking Mental Health Services (IASMHS) [77] on a 5-point rating scale. It comprises 24 items. Its 3 dimensions—“psychological openness,” “help-seeking propensity,” and “indifference to stigma”—are represented with 8 items. Subscale scores range from 0 to 32. Higher scores indicate more positive attitudes.

The Universal Stigma Scale (USS) [78] was used for stigma measurement. It consists of 11 statements in 2 subscales (“blame/personal responsibility”: 5 items; “impairment/distrust”: 6 items). The extent of agreement with these statements is indicated on a 5-point Likert scale. Means were calculated for each of the 2 subscales. Lower scores indicate higher stigmatization.

Transportation (ie, the immersiveness of the stories presented in the videos) was measured using an adapted version of the Transportation Scale–Short Form [79]. Adjustments were made to suit the medium of the narratives (ie, video material in contrast to written stories). Our adapted version contained 5 items on a 7-point Likert scale.

Video acceptability was measured using a translated and adapted 4-item version of the acceptability and likability scale used by Gaudiano et al [45]. In total, 3 items measured overall likability, comprehensibility, and interestingness of the videos on a 5-point rating scale.

### Statistical Analysis

Sociodemographic, screening, and outcome data were first analyzed descriptively. Intervention effects on potential professional help seeking (primary outcome) and secondary outcomes in the total sample (ie, across all MH problems and across participants with and without actual help seeking as reported in the screening) were analyzed via analyses of covariance (ANCOVAs) at an α level of *P*<.05. In addition to the intervention group, the models included age as a covariate, the participants’ actual help seeking (fixed effects), and the 5 MH problems (random effects) as control variables. The results of the main ANCOVA in the total sample are presented in the Results section.

Subgroup ANCOVAs were conducted for each of the 5 MH problems separately. In this case, the respective screening scores (GAD-7, PHQ-9, WCS, number of NSSI events during the last year, and AUDIT-C) were included as additional covariates. Subgroup analyses were further conducted for cases with and without actual help seeking in the total sample and within each of the 5 MH issue groups.

In case of significant (*P*<.05) and trend ANCOVA effects, pairwise group comparisons were conducted using 2-tailed *t* tests. All tests were 2-sided with an α level of 5%. Mean differences (MDs) adjusted for covariates are reported in the Results section.

An a priori power analysis was conducted using G*Power (Heinrich-Heine-Universität Düsseldorf) [80]. Under the assumption of a medium effect size (*f*=0.25), a minimum of 240 participants (80 per condition) were needed to test the expected effect within each of the 5 MH problems via ANCOVAs with a significance criterion of α=.05 and a power of 90%. Statistical analyses were performed using R (version 4.3; R Foundation for Statistical Computing) [81] and SPSS (version 28; IBM Corp) [82]. R was also used to generate the random allocation sequence. Authors involved in data analysis and interpretation were not blinded with respect to the assigned experimental conditions.

### Deviations From the Protocol

In the beginning of recruitment, the upper age limit was raised from 25 years originally to 29 years due to the aforementioned findings of previous research.

### Ethical Considerations

Ethics approval was obtained from Ethics Committee I of the Heidelberg Medical Faculty on July 27, 2020 (protocol S378/2020). The procedures were in accordance with the Helsinki Declaration of 1975, as revised in 2000. All participants received information about the study’s aims, scope, procedures, data processing, and data storage on the study website in written form. Furthermore, all participants received contact information if they wished to clarify questions via telephone or email. Only participants who provided their informed consent through a web-based checkbox were eligible for participation. Participants were able to opt out of the study at any time by closing the study website, which they were informed of before their participation. Participants were offered to take part in an optional gift card lottery at the end of the study (100 gift cards of €20 [US $21.58] each). If they were interested in the lottery, they could enter their email address. Email addresses were stored separately from other study data and user IDs to ensure anonymity. All other data were collected and are reported anonymously. Thus, this study does not contain any individual data of identifiable participants.

## Results

### Sample Characteristics

Figure 1 shows the flow of participants. Of the 2208 participants who were randomized to 1 of the 15 conditions, 472 (21.38%) were excluded because their time spent on the video pages fell below the durations of the videos they were assigned to, indicating that they did not view the entire videos. Of the remaining 1736 participants, 342 (19.7%) were excluded due to incomplete data (ie, they did not complete all the relevant scales that the study entailed [beginning with informed consent up to and including the last acceptability item]). Our final sample consisted of 1394 youths aged 14 to 29 years (mean 20.97, SD 3.67 years). Sociodemographic and screening results are shown in Table 1.

**Table 1:** Sociodemographic characteristics and screening results (n=1394).

Characteristics			Values
Age (years), mean (SD)			20.97 (3.67)
**Gender, n (%)**			
	Girl and woman	1109 (79.6)	
	Boy and man	254 (18.2)	
	Nonbinary	31 (2.2)	
**Actual help seeking, n (%)**			
	None	770 (55.2)	
	Current	273 (19.6)	
	Past	351 (25.2)	
**Knew someone with MH^a^ problems, n (%)**			
	Yes	1285 (92.2)	
	No	109 (7.8)	
**Education (highest secondary school diploma), n (%)**			
	Still in school	264 (18.9)	
	No diploma	0 (0)	
	Lower secondary school diploma (*Hauptschulabschluss*)	8 (0.6)	
	Intermediate secondary school diploma (*Realschulabschluss*)	89 (6.4)	
	Higher secondary school diploma (*Abitur*)	1016 (72.9)	
	Other	17 (1.2)	
**Migration background, n (%)**			
	None	1104 (79.2)	
	First generation or not born in Germany	64 (4.6)	
	Second generation	226 (16.2)	
**GAD-7^b^ score, mean (SD)**			8.38 (5.00)
	Minimal or no anxiety (0-4)	370 (26.5)	
	Mild (5-9)	501 (35.9)	
	Moderate (10-14)	330 (23.7)	
	Severe (≥15)	193 (13.9)	
**PHQ-9^c^ score, mean (SD)**			9.56 (6.07)
	Minimal or no depression (0-4)	333 (23.9)	
	Mild (5-9)	430 (30.9)	
	Moderate (10-14)	347 (24.9)	
	Moderately severe (15-19)	171 (12.3)	
	Severe (≥20)	113 (8.1)	
**WCS^d^ score, mean (SD)**			34.50 (24.65)
	High risk (≥57)	273 (19.6)	
	Low risk (<57)	1121 (80.4)	
**SITBI-G^e^ score, mean (SD)**			
	Lifetime NSSI^f^	479 (34.4)	
	12-month NSSI	265 (19.0)	
	Number of NSSI events (previous 12 months; lifetime NSSI sample [n=479])	11.98 (45.36)	
	Number of NSSI events (previous 12 months; 12-month NSSI sample [n=265])	21.66 (59.29)	
	Age of first NSSI (n=479)	14.20 (3.01)	
	Age of last NSSI – age of first NSSI (n=475)^g^	3.92 (3.71)	
**AUDIT-C^h^ score, mean (SD)**			2.51 (2.08)
	Abstinent (0)	319 (22.9)	
	Moderate (1-3)	645 (46.3)	
	Hazardous (≥4)	430 (30.9)	

^a^MH: mental health.

^b^GAD-7: 7-item Generalized Anxiety Disorder Scale.

^c^PHQ-9: 9-item Patient Health Questionnaire.

^d^WCS: Weight Concerns Scale.

^e^SITBI-G: German version of the Self-Injurious Thoughts and Behaviors Interview.

^f^NSSI: nonsuicidal self-injury.

^g^We excluded 4 cases in “Age of last NSSI – age of first NSSI” due to invalid values (age of first NSSI>age of last NSSI).

^h^AUDIT-C: Alcohol Use Disorders Identification Test for Consumption.

A total of 79.56% (1109/1394) of the sample identified as woman or girl, and 44.76% (624/1394) were help seekers (ie, they used professional MH services at the time of or before data collection). On average, the youth were moderately anxious (mean GAD-7 score 8.38, SD 5.00) and depressed (mean PHQ-9 score 9.56, SD 6.07). While 22.88% (319/1394) reported abstinence in the AUDIT-C, 30.85% (430/1394) indicated hazardous alcohol consumption. A total of 19.58% (273/1394) were at high risk of developing an eating disorder according to the WCS. One-third (479/1394, 34.36%) of the sample reported a lifetime history of NSSI according to the Self-Injurious Thoughts and Behaviors Interview, with a 12-month prevalence rate of 19.01% (265/1394).

### Intervention Effects

The main results are presented in Table 2.

**Table 2:** Analysis of covariance results and pairwise comparisons for primary outcomes (total sample; n=1394)^a^.

			Total, mean (SD)		CG^b^ (n=554), mean (SD)		Intervention 1 (n=410), mean (SD)		Intervention 2 (n=430), mean (SD)		*F* test (*df*=2, 1385)	*P* value	Pairwise comparisons
**Potential help seeking (GHSQ^c^)^d^**													
	Professional maximum	4.74 (1.81)		4.65 (1.88)		4.82 (1.74)		4.76 (1.79)		0.99		.37	—^e^
	Informal maximum	5.86 (1.38)		5.87 (1.37)		5.73 (1.43)		5.95 (1.35)		3.75		.02	Intervention 2>intervention 1
	None	3.07 (2.01)		3.08 (2.02)		3.20 (1.99)		2.94 (2.01)		2.68		.07	—
**Stigma (USS^f^)^g^**													
	Blame	4.47 (0.64)		4.41 (0.68)		4.50 (0.65)		4.50 (0.58)		3.25		.04	Intervention 1; intervention 2>CG
	Distrust	3.94 (0.79)		3.84 (0.79)		3.97 (0.79)		4.02 (0.78)		8.01		<.001	Intervention 1; intervention 2>CG
**Help-seeking attitudes (IASMHS^h^)^g^**													
	Psychological openness	21.20 (4.82)		21.05 (4.84)		21.65 (4.70)		20.97 (4.89)		1.67		.19	—
	Help seeking propensity	20.95 (5.29)		20.78 (5.41)		21.04 (5.14)		21.09 (5.27)		0.48		.62	—
	Indifference to stigma	23.37 (6.31)		23.83 (6.06)		23.02 (6.51)		23.13 (6.40)		3.18		.04	CG>intervention 1
**Video acceptability and transportation^d^**													
	General likability	3.94 (0.81)		3.85 (0.78)		4.10 (0.79)		3.90 (0.84)		12.20		<.001	Intervention 1>CG; intervention 2
	Comprehensibility	4.82 (0.45)		4.79 (0.50)		4.82 (0.45)		4.85 (0.40)		2.01		.13	—
	Interestingness	3.86 (0.96)		3.84 (0.93)		3.98 (0.94)		3.76 (0.99)		6.39		.002	Intervention 1>CG; intervention 2
	Transportation (TS-SF^i^)	4.49 (1.24)		4.53 (1.21)		4.58 (1.25)		4.37 (1.27)		4.23		.02	CG; intervention 1>intervention 2

^a^Results controlled for help seeking (fixed factor), mental health issue (random factor), and age (covariate).

^b^CG: control group.

^c^GHSQ: General Help Seeking Questionnaire.

^d^Higher scores represent a greater level of agreement.

^e^Pairwise comparisons were conducted in case of significant or trend analysis of covariance effects. Empty cells indicate that pairwise comparisons were not conducted due to the analysis of covariance results.

^f^USS: Universal Stigma Scale.

^g^Higher scores represent more positive attitudes toward mental health issues and help seeking.

^h^IASMHS: Inventory of Attitudes Toward Seeking Mental Health Services.

^i^TS-SF: Transportation Scale–Short Form.

**Figure 2:**
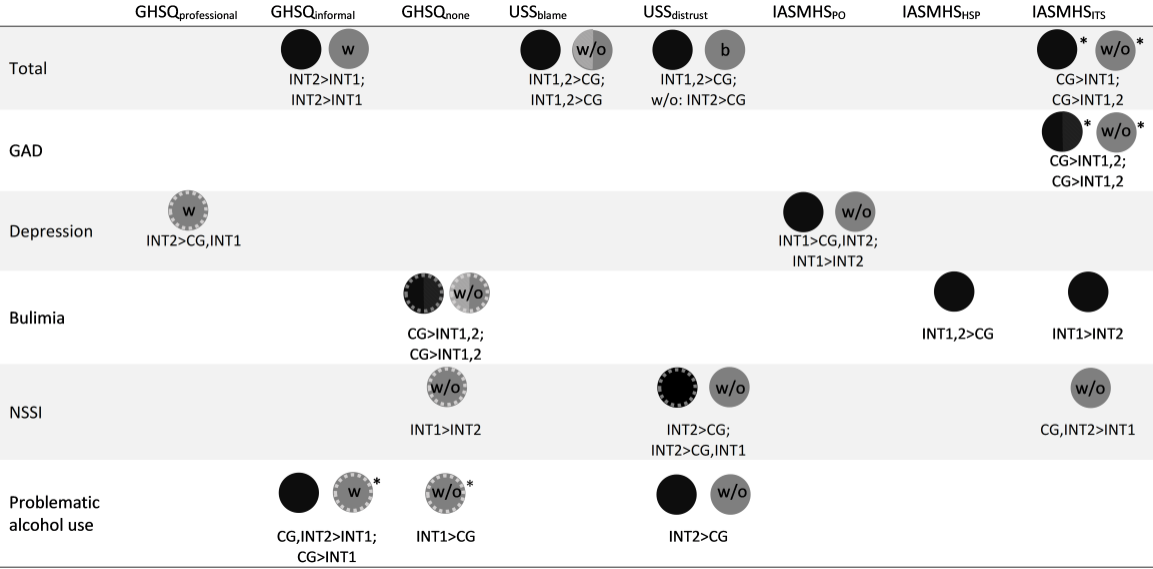
Analysis of covariance (ANCOVA) results (overview). Black spots represent group effects on the total sample (with and without previous help seeking), gray spots represent group effects on one or both subsamples, dotted circles indicate nonsignificant trend group differences with *P*<.06 in the ANCOVAs, and striped areas indicate nonsignificant trend group differences with *P*<.06 in the post hoc comparisons. Asterisks indicate more favorable outcomes for the control group (CG) in comparison to both intervention groups. In the GHSQ, higher scores represent a greater level of agreement. In the other outcome measures, higher scores represent more positive attitudes toward MH issues and help seeking. b: both subsamples (with and without previous help seeking); GAD: generalized anxiety disorder; GHSQ: General Help Seeking Questionnaire; HSP: help seeking propensity; IASMHS: Inventory of Attitudes Toward Seeking Mental Health Services; INT1: intervention 1; INT2: intervention 2; ITS: indifference to stigma; MH: mental health; NSSI: nonsuicidal self-injury; PO: psychological openness; USS: Universal Stigma Scale; w/o: without previous help seeking; w: with previous help seeking.

Figure 2 summarizes the results of the overall efficacy and the MH issue–specific subgroup analyses graphically. Specific results of the subgroup analyses can be found in Multimedia Appendices 1, 2, and 3.

### Primary Outcome: Potential Professional Help Seeking (GHSQ)

On the 7-point scale of the GHSQ, most participants (1046/1394, 75.04%) selected a score of ≥4 (CG: 409/554, 73.8%; intervention 1: 316/410, 77.1%; intervention 2: 321/430, 74.7%). In total, 19.23% (268/1394; CG: 105/554, 19%; intervention 1: 74/410, 18%; intervention 2: 89/430, 20.7%) of participants reported a score of 7 (“extremely likely”), whereas 6.74% (94/1394; CG: 47/554, 8.5%; intervention 1: 24/410, 5.9%; intervention 2: 23/430, 5.3%) responded with a score of 1 (“extremely unlikely”). Across all MH problems, no statistically significant group main effect was found on potential professional help seeking (*F*_2,1385_=0.99; *P*=.37; Table 2).

### Secondary Outcomes

#### Potential Informal Help Seeking (GHSQ)

For informal sources of support, most participants (1190/1394, 85.37%) selected a score of ≥5 on the 7-point scale (CG: 478/554, 86.3%; intervention 1: 338/410, 82.4%; intervention 2: 374/430, 87%). For 43.69% (609/1394) of the participants, informal help seeking was “extremely likely,” with a selected score of 7 (CG: 244/554, 44%; intervention 1: 161/410, 39.3%; intervention 2: 204/430, 47.4%), whereas a minority of 1% (14/1394; CG: 4/554, 0.7%; intervention 1: 7/410, 1.7%; intervention 2: 3/430, 0.7%) responded with a score of 1 (“extremely unlikely”). In the total sample, significant group differences were found regarding informal help seeking (*F*_2,1385_=3.75; *P*=.02), with intervention 2 showing a significantly higher mean score than intervention 1 (adjusted MD=0.25; *P*=.007; Table 2). In the subsample of help seekers across MH problems, the same pattern was observed (*F*_2,616_=3.21; *P*=.04; adjusted MD=0.37; *P*=.01; Multimedia Appendix 1). A significant group effect was also found for the total sample in the problematic alcohol use conditions (*F*_2,273_=3.51; *P*=.03; Multimedia Appendix 2). Both the CG (adjusted MD=0.42; *P*=.02) and intervention 2 (adjusted MD=0.41; *P*=.03) had greater mean scores than intervention 1.

#### No Potential Help Seeking (GHSQ)

With regard to no intention of seeking help with any of the potential sources listed in the GHSQ (“I would not seek help from anyone” item), almost half (674/1394, 48.35%) of participants selected a score of 1 or 2 (1=“extremely unlikely”; CG: 264/554, 47.7%; intervention 1: 185/410, 45.1%; intervention 2: 225/430, 52.3%), whereas 15.42% (215/1394) responded with a score of 6 or 7 (7=“extremely likely”; CG: 89/554, 16.1%; intervention 1: 68/410, 16.6%; intervention 2: 58/430, 13.5%). There were no statistically significant group differences in the total sample (*P*=.07; Table 2). However, there were trends for group differences in some of the MH issue subgroups (Multimedia Appendices 2 and 3 and Figure 2).

#### Public Stigma: Blame and Personal Responsibility (USS)

With regard to the USS blame and personal responsibility subscale, statistically significant group differences were found in the total sample (*F*_2,1385_=3.25; *P*=.04; Table 2) and in non–help seekers across MH problems (*F*_2,762_=3.21; *P*=.04; Multimedia Appendix 1). In the total sample, both intervention 1 and intervention 2 had significantly greater means compared to the CG (intervention 1>CG: adjusted MD=0.084 and *P*=.03; intervention 2>CG: adjusted MD=0.085 and *P*=.03). In the subgroup of non–help seekers, there was a significant difference between intervention 2 and the CG (adjusted MD=0.13; *P*=.02). Further subgroup analyses revealed no additional differences between experimental conditions. It should be noted that blame and personal responsibility data distributions were heavily skewed to the left (total sample: skew=−1.58). As logarithmic, natural logarithm, square root, and reciprocal transformations did not normalize the distributions, we decided to perform ANCOVAs using the untransformed blame data. Therefore, results should be interpreted with caution.

#### Public Stigma: Impairment and Distrust (USS)

For the USS distrust subscale, ANCOVAs revealed statistically significant group differences in the total sample (*F*_2,1385_=8.01; *P*<.001; Table 2) in both help seekers (*F*_2,616_=4.39; *P*=.01) and non–help-seekers across MH problems (*F*_2,762_=3.74; *P*=.02; Multimedia Appendix 1). Moreover, statistically significant group differences were found in the total problematic alcohol use subsample (*F*_2,273_=4.49; *P*=.01; Multimedia Appendix 2) and its subgroup of non–help seekers (*F*_2,144_=4.00; *P*=.02; Multimedia Appendix 3). In the NSSI subgroup of non–help seekers, a significant group main effect was observed (*F*_2,160_=4.50; *P*=.01; Multimedia Appendix 3). Across MH problems, both in the total sample (intervention 1>CG: adjusted MD=0.13 and *P*=.005; intervention 2>CG: adjusted MD=0.17 and *P*<.001) and the subsample of help seekers (intervention 1>CG: adjusted MD=0.16 and *P*=.02; intervention 2>CG: adjusted MD=0.17 and *P*=.01), significantly larger means in both interventions as compared to the CG were observed. Among participants without previous help seeking across MH problems, post hoc comparisons only revealed a statistically significant difference between intervention 2 and the CG (adjusted MD=0.16; *P*=.007). In the NSSI subgroup of non–help seekers, intervention 2 differed significantly from both the CG (adjusted MD=0.36; *P*=.005) and intervention 1 (adjusted MD=0.31; *P*=.02). For problematic alcohol use, in both the total sample and the subsample of non–help seekers, significant post hoc differences between intervention 2 and the CG (MD for the total=0.32 and *P*=.003; MD for those without previous help seeking=0.42 and *P*=.006) were found.

#### Psychological Openness (IASMHS)

No statistically significant group main effect on the IASMHS psychological openness subscale was found in the total sample (Table 2). Significant effects were found in the total depression sample (*F*_2,255_=4.59; *P*=.01; Multimedia Appendix 2) and its subgroup of non–help seekers (*F*_2,138_=4.20; *P*=.02; Multimedia Appendix 3). In the total depression sample, intervention 1 showed a greater mean in comparison to the CG (adjusted MD=1.38; *P*=.046) and intervention 2 (adjusted MD=2.24; *P*=.003). In the subsample of non–help seekers in the depression conditions, intervention 1 was found to have a greater mean than intervention 2 (adjusted MD=2.75; *P*=.004), but no significant difference was found with the CG (adjusted MD=1.55; *P*=.09). No significant group main effects were observed in the other subsamples.

#### Help Seeking Propensity (IASMHS)

In the total sample, no significant group main effect was found for the IASMHS help seeking propensity subscale (Table 2). Subgroup analyses revealed significant differences in the total bulimia sample (*F*_2,271_=3.27; *P*=.04), where both intervention 1 (adjusted MD=1.51; *P*=.03) and intervention 2 (adjusted MD=1.40; *P*=.04) showed larger means than the CG (Multimedia Appendix 2). No further group differences were found in the other subsamples.

#### Indifference to Stigma (IASMHS)

For the IASMHS indifference to stigma subscale, differential group main effects were found in the total sample (*F*_2,1385_=3.18; *P*=.04; Table 2), in the subsample of non–help seekers (*F*_2,762_=3.74; *P*=.02; Multimedia Appendix 1), in the total (*F*_2,288_=3.22; *P*=.04; Multimedia Appendix 2) and non–help-seeking (*F*_2,176_=4.48; *P*=.01; Multimedia Appendix 3) GAD samples, in the total bulimia sample (*F*_2,271_=3.45; *P*=.03; Multimedia Appendix 2), and in the NSSI subsample of non–help seekers (*F*_2,160_=3.23; *P*=.04; Multimedia Appendix 3). Across MH problems, the CG showed a larger mean than intervention 1 in the total sample (adjusted MD=0.97; *P*=.02), whereas a greater mean score in the CG compared to those of both intervention 1 (adjusted MD=1.20; *P=*.02) and intervention 2 (adjusted MD=1.15; *P*=.02) was found in the subsample without previous help seeking. A similar pattern emerged in the total GAD sample and its subsample of non–help seekers, where the CG’s means were significantly larger in comparison to those of intervention 1 (adjusted MD=2.09; *P*=.02) in the total sample and of both intervention 1 (adjusted MD=2.57; *P*=.02) and intervention 2 (adjusted MD=2.91; *P*=.008) among non–help seekers. In the total bulimia sample, intervention 1 had a significantly higher mean than intervention 2 (adjusted MD=2.26; *P*=.009), whereas both the CG (adjusted MD=2.10; *P*=.04) and intervention 2 had greater means than intervention 1 (adjusted MD=2.59; *P=*.02) in the NSSI subsample of non–help seekers.

#### Video Acceptability and Transportation

In the total sample, most participants (1041/1394, 74.68%) rated the videos with a score of “4” (705/1394, 50.57%) or “5” (336/1394, 24.1%) on the overall likability item. Regarding comprehensibility, 83.93% (1170/1394) rated the videos as “very comprehensible” (“5” on the 5-point scale), whereas 14.13% (197/1394) assigned them a score of “4.” With respect to the videos’ interestingness, the responses were distributed across the 5-point scale as follows: 27.4% (382/1394) of participants gave a rating of “5,” a total of 41.61% (580/1394) gave the videos a rating of “4,” a total of 22.02% (307/1394) assigned them a score of “3,” and 7.32% (102/1394) gave them a rating of “2.” A minority of participants (23/1394, 1.65%) rated the videos with a score of “1” on the interestingness scale.

In the total sample (Table 2), the intervention 1 videos were rated as generally more likable (*F*_2,1385_=12.20; *P*<.001; intervention 1>CG: adjusted MD=0.25 and *P*<.001; intervention 1>intervention 2: adjusted MD=0.20 and *P*<.001) and interesting (*F*_2,1385_=6.39; *P*=.002; intervention 1>CG: adjusted MD=0.06 and *P*=.02; intervention 1>intervention 2: adjusted MD=0.07 and *P*<.001) in comparison to those of the CG and intervention 2. The groups did not differ significantly in video comprehensibility (*F*_2,1385_=2.01; *P*=.13). Participants felt more “transported” into the videos’ narratives in the CG and intervention 1 as compared to participants in intervention 2 (*F*_2,1385_=4.23; *P*=.02; CG>intervention 2: adjusted MD=0.17 and *P*=.03; intervention 1>intervention 2: adjusted MD=0.23 and *P*=.006; Table 2). Most subgroup analyses revealed either similar patterns with regard to general likability and interestingness (eg, total help seekers, total GAD sample, and GAD non–help seekers) or no significant differences (eg, GAD help seekers, bulimia help seekers, all depression samples, and all alcohol use samples; Multimedia Appendices 1-3). In the cases of bulimia (total and non–help-seeking subsamples; Multimedia Appendices 2 and 3) and NSSI (Multimedia Appendix 2), different patterns emerged. In the total bulimia sample, the videos of both the CG and intervention 1 scored significantly higher on the interestingness scale than those of intervention 2 (*F*_2,271_=4.49; *P*=.01; CG>intervention 2: adjusted MD=0.33 and *P*=.02; intervention 1>intervention 2: adjusted MD=0.44 and *P*=.005). In the total NSSI sample, the videos of both intervention 1 and intervention 2 were rated as significantly more likable than those of the CG (*F*_2,277_=10.31; *P*<.001; intervention 1>CG: adjusted MD=0.51 and *P*<.001; intervention 2>CG: adjusted MD=0.29 and *P*=.008).

## Discussion

### Principal Findings

This study developed and tested the short-term effectiveness of 2 brief video-based strategies targeted at adolescents and young adults (aged 14 to 29 years) aiming to foster potential professional help seeking (main outcome) and related attitudes for 5 MH problems. In the total sample, we did not find effects of either intervention 1 (psychoeducation) or intervention 2 (positive outcome expectancies) on our primary outcome. However, significant group effects were found with respect to potential informal help seeking, stigma toward others, and indifference to stigma in the total sample. While both intervention groups showed more favorable attitudes than the CG with regard to public stigma, this did not translate to participants’ own indifference to stigma. In this case, the CG showed significantly more positive attitudes in comparison to intervention 1. However, this finding was not apparent in the MH issue–specific subgroup analysis with the exception of GAD. Unintended adverse effects of MH interventions have been reported in previous research [83-85], which underlines the need for thorough evaluations of such interventions before their public dissemination. Accordingly, we would advise against the implementation of our GAD interventions at the current stage and would recommend the development and evaluation of other tailored strategies for this MH problem.

With regard to informal help, participants in intervention 2 showed a greater willingness to approach friends, family members, or romantic partners for help than participants in intervention 1. This might have been due to the interventions’ design as intervention 2 explicitly depicted improvements in social relationships after the main characters in the videos had sought professional support. Overall, the videos were well accepted and rated as quite interesting, with some room for improvement and with the videos of intervention 1 outperforming those of the other 2 conditions. All videos were, on average, rated as very comprehensible, and no significant group differences were observed in this regard. Interestingly, participants felt more transported into the narratives in the CG and intervention 1 as compared to those in intervention 2. As intervention 2 followed a narrative approach, continuing Paul and Paula’s vignette stories, this was surprising. However, as previously stated, intervention 1 was generally more liked and viewed as more interesting in comparison to intervention 2. The animated and fictional third-person approach of intervention 2 seemed to not have sparked as much interest in participants as the facts presented in intervention 1. While we aimed to increase identification with our main characters through the alignment of their genders with those of the participants, intervention 2 might have been insufficient with regard to the perceived “realness” of the story and the characters, which has been identified as crucial for the formation of narrative transportation and, in turn, attitudes and intentions [86]. The rather optimistic portrayal of the help-seeking process might have contributed to a lack of perceived authenticity in this sample as well. Furthermore, implicit MH statements in the videos’ scenarios could have been more fruitful. For instance, the viewer could have watched directly how Paul and Paula discussed their issues with a psychotherapist rather than having a narrator describe the situation to them. These types of videos have been associated with improved health literacy and more beneficial attitudes toward cervical cancer [87], and their application to the field of MH would be interesting.

We further observed differential outcomes with respect to the assigned MH problems and participants’ actual help-seeking status. While the CG outperformed either intervention 1 (total) or both interventions (subgroup of non–help seekers) for GAD with regard to stigma indifference and no further outcomes were found for GAD, different patterns emerged in the other MH issue groups. Results were mixed, where both intervention 1 and intervention 2 outperformed the other conditions in some of the outcomes but not in others (eg, in depression), or a clearer tendency toward the superiority of one of the interventions emerged (eg, in NSSI). In summary, our results point toward the usefulness of tailored interventions with regard to MH issue type and previous help-seeking experiences of potential target groups. Our finding that different strategies might work differently for each of the 5 MH problems included in our study is in accordance with those of previous research. For example, Ebneter and Latner [78] found varying stigmatizing attitudes among different MH problems. The participants in their study blamed a vignette character with an eating disorder more for their condition than a character with depression, whereas the latter was regarded as more impaired. Our finding that a destigmatizing and psychoeducational intervention such as intervention 1 might work better for bulimia fits their recommendation to target stigmatizing attitudes toward specific MH problems [78]. Similarly, alcohol dependency and self-endangering behaviors were perceived as particularly dangerous in a Swiss vignette study [88]. The humanizing depiction of our NSSI and problematic alcohol use characters within a framework of close supportive relationships, which improved in quality through psychotherapy, might have been a relevant factor for reduced distrust scores in the intervention 2 condition as compared to the CG and in the case of non–help seekers in the NSSI condition as compared to intervention 1 as well. This approach might be advantageous to reduce public stigma regarding MH problems that are viewed as particularly dangerous. Decisions for one or another interventional strategy may also depend on the specific goal and targeted outcome. While more research is needed, our study provides preliminary evidence for the tailored strategies suggested in Table 3.

**Table 3:** Mental health (MH) problem–specific intervention recommendations based on the study results^a^.

MH problem	Recommended intervention		Aims
	Psychoeducation (intervention 1)	Outcome expectancies (intervention 2)	
All MH problems	?	✓	Antistigma (others), motivate informal help seeking or peer support
GAD^b^	X	X	—^c^
Depression	?	?	Intervention 1: normalize MH-related topics; intervention 2: motivate previous help seekers to reseek professional support in case of relapse
Bulimia	✓	?	Antistigma (self), strengthen help seeking propensity
NSSI^d^	X	✓	Antistigma (others)
Problematic alcohol use	X	✓	Antistigma (others)

^a^Check marks (✓) represent recommended use of an intervention; crosses (X) represent advice against the use of an intervention; and question marks (?) represent inconclusive results and, therefore, no clear recommendation. Recommendations are solely based on the results of this study.

^b^GAD: generalized anxiety disorder.

^c^No recommended use.

^d^NSSI: nonsuicidal self-injury.

### Limitations

One limitation of this study lies within the sole investigation of effects on hypothetical intentions and attitudes instead of actual help-seeking behavior. While intentions are substantially associated with behavior and provide valuable insights, they do not translate directly to behavior change [49]. Moreover, only short-term effects were investigated. Previous studies on short video-based interventions have demonstrated sustaining destigmatizing effects for 1 [40] and 5 months after their delivery [43]. While potential long-term effects of our interventions in particular remain unknown and might be investigated in the future, the current state of research points toward potentially impactful long-term effects of low-threshold microinterventions. Related to this, we did not investigate dose-response effects. Research on optimal doses (ie, durations, frequencies, and amounts of intervention components) needed for sustainable change through microinterventions and interventions in general is crucial for well-founded recommendations for or against specific interventions [89].

Just as in other microintervention studies among youth [39], group differences in our study were small. However, the potential high reach of easily accessible, low-threshold interventions such as the ones evaluated in this study is apparent in the final sample size of 1394. As it depends on both effectiveness and reach, this allows for a comparably high public health impact [23,24]. We also included trend effects in our overview (Figure 2), which should be interpreted with caution. However, these findings might be useful to inform the planning of subsequent research in this field.

Future research should focus on improvements in the effectiveness of microinterventions. One approach could be the investigation of interventional framing. In a Japanese study on depression, loss-framed messages (ie, emphasizing negative consequences of refraining from help seeking) had a greater impact on help-seeking intentions than gain-framed (positive consequences of help seeking) or neutral (eg, prevalence rates) messages as well as unformatted, plain-text messages in middle-aged adults [90]. Thus, it might be interesting to conduct future studies on the effects of video-based microinterventions with differently framed messaging as the videos in this study emphasized potential gains of help seeking rather than potential losses of help seeking restraint.

Furthermore, our sample showed, on average, a high level of education, very little public stigma, a pronounced willingness to seek help, and a high rate of actual professional help seeking (624/1394, 44.76%), which limits the generalizability of our findings. More than 90% (1285/1394, 92.18%) of our sample knew someone with MH problems. While we were careful not to recruit MH experts, such as university students of medicine and psychology, youth with a personal interest in MH-related topics seemed to have been more inclined to participate. Related to this, we aimed for a community youth sample rather than a clinical sample. The scenarios that our items referred to were hypothetical and did not necessarily reflect participants’ own experiences due to the random assignment to 1 of the 5 MH problems. A similar approach with targeted interventions according to youth’s actual MH status and more individualized elements with regard to gender-related [91] and cultural [92,93] aspects could be promising in future research. Thus, upcoming studies should strive to align their research objectives more closely with the characteristics and needs of the selected target groups.

Finally, we did not include a comprehension check to assess participants’ understanding of and engagement with the content presented in the videos. While we accounted for the time participants spent on the video pages and only included participants with sufficient durations of stay in the final analyses, they may not have fully comprehended or attended to the video material. The substantial number of excluded participants who completed the study without meeting the time threshold (472/2208, 21.38%) underlines this potential issue. Thus, future studies should address this limitation by including comprehension checks to improve the robustness of the findings.

### Conclusions

The low uptake of professional MH services in youth reflects the need for appropriate strategies to facilitate professional help seeking. This study investigated the effectiveness of 2 short video-based strategies targeted at youth (aged 14 to 29 years) on potential professional help seeking and related attitudes for 5 MH problems. While we did not find intervention effects on potential professional help seeking (with the exception of previous help seekers in the depression conditions), differential intervention effects depending on each MH problem and participants’ actual help-seeking status were found in our secondary outcomes, such as public stigma. Our study results can be used to inform the development of new antistigma interventions, which, based on our results, we would recommend tailoring to specific MH problems, target groups, and outcomes. While group differences were small, such low-threshold interventions can be easily disseminated and, therefore, hold potential for a high reach and, thus, a meaningful impact at the population level. More research is needed for more robust and generalizable recommendations.

## Appendix

Multimedia Appendix 1 Separate analysis of covariance results and pairwise comparisons for participants with and without previous help-seeking experience.

Multimedia Appendix 2 Separate analysis of covariance results and pairwise comparisons for outcomes per mental health problem.

Multimedia Appendix 3 Separate analysis of covariance results and pairwise comparisons for participants with and without previous help-seeking experience per mental health problem.

Multimedia Appendix 4 CONSORT-eHEALTH Checklist V 1.6.1.

## Abbreviations

ANCOVA: analysis of covariance
AUDIT-C: Alcohol Use Disorders Identification Test for Consumption
CG: control group
CONSORT-EHEALTH: Consolidated Standards of Reporting Trials of Electronic and Mobile Health Applications and Online Telehealth
GAD: generalized anxiety disorder
GAD-7: 7-item Generalized Anxiety Disorder Scale
GHSQ: General Help Seeking Questionnaire
HAPA: Health Action Process Approach
IASMHS: Inventory of Attitudes Toward Seeking Mental Health Services
MD: mean difference
MH: mental health
NSSI: nonsuicidal self-injury
PHQ-9: 9-item Patient Health Questionnaire
RCT: randomized controlled trial
USS: Universal Stigma Scale
WCS: Weight Concerns Scale

## Data Availability

Individual participant data underlying the results presented in this publication (text, tables, figures, and appendices) and statistical code will be shared during the period of 3 months to 5 years following publication of the paper with researchers who provide a methodologically sound proposal to the corresponding author.
