# Supplementary material for: The Impact of Video-Based Microinterventions on Attitudes Toward Mental Health and Help Seeking in Youth: Web-Based Randomized Controlled Trial
Source: J Med Internet Res. 2024 Apr 24;26:e54478. doi: 10.2196/54478 (PMC11079770; doi:10.2196/54478)
Supplement: Multimedia Appendix 1 [file jmir_v26i1e54478_app1.doc]

**Multimedia Appendix**

# **Appendix 1: Separate ANCOVA results and pairwise comparisons for participants with and without prior help-seeking experience.**

## **Table S1. ANCOVA results and pairwise comparisons for outcomes (all mental health issues, participants *with* prior help-seeking experience only).**

| Subsample *with* prior help-seeking | | | | | | | |
| --- | --- | --- | --- | --- | --- | --- | --- |
|  | total  *N*=624 | CG  *n*=246 | INT1  *n*=184 | INT2  *n*=194 | *F* (2,616) | *p* | Pairwise comparisons |
| Potential help-seeking (GHSQ)^a^ |  | | | | | | |
| Professional max. *M (SD)* | 5.29 (1.68) | 5.21 (1.75) | 5.35 (1.62) | 5.35 (1.64) | .32 | .728 |  |
| Informal max. *M (SD)* | 5.72 (1.45) | 5.72 (1.49) | 5.54 (1.50) | 5.89 (1.34) | 3.21 | .041 | INT2 > INT1 |
| None *M (SD)* | 3.00 (2.07) | 3.02 (2.09) | 3.08 (2.04) | 2.92 (2.06) | .41 | .662 |  |
| Stigma (USS)^b^ |  | | | | | | |
| Blame *M (SD)* | 4.56 (.57) | 4.54 (.57) | 4.57 (.57) | 4.56 (.60) | .36 | .696 |  |
| Distrust *M (SD)* | 4.03 (.78) | 3.93 (.76) | 4.08 (.80) | 4.11 (.79) | 4.39 | .013 | INT1, INT2 > CG |
| Help-seeking attitudes (IASMHS)^b^ |  | | | | | | |
| Psychological Openness *M (SD)* | 21.87 (4.62) | 21.68 (4.84) | 22.40 (4.28) | 21.61 (4.64) | 1.55 | .212 |  |
| Help-seeking propensity *M (SD)* | 22.39 (5.38) | 22.19 (5.40) | 22.51 (5.47) | 22.54 (5.29) | .20 | .819 |  |
| Indifference to stigma *M (SD)* | 23.45 (6.70) | 23.62 (6.56) | 23.08 (6.88) | 23.58 (6.71) | .59 | .554 |  |
| Video acceptability and transportation^a^ | | | | | | | |
| General likability *M (SD)* | 3.89 (.79) | 3.81 (.77) | 4.05 (.77) | 3.82 (.82) | 5.89 | .003 | INT1 > CG, INT2 |
| Comprehensibility *M (SD)* | 4.82 (.44) | 4.79 (.46) | 4.85 (.39) | 4.83 (.44) | 1.03 | .358 |  |
| Interestingness *M (SD)* | 3.84 (.96) | 3.83 (.93) | 3.98 (.97) | 3.72 (.97) | 3.76 | .024 | INT1 > INT2 |
| Transportation (TS-SF) *M (SD)* | 4.59 (1.29) | 4.62 (1.28) | 4.71 (1.27) | 4.44 (1.32) | 2.43 | .089 |  |

*Note.* *CG* = control group; *GHSQ* = General Help Seeking Questionnaire; *IASMHS* = Inventory of Attitudes toward Seeking Mental Health Services; *INT1* = intervention 1; *INT2* = intervention 2; *TS-SF* = Transportation Scale - Short Form*; USS* = Universal Stigma Scale. ^a^ Higher scores represent a greater level of agreement. ^b^ Higher scores represent more positive attitudes towards mental health issues and help-seeking. Results controlled for MH issue (random factor) and age (covariate).

## **Table S2. ANCOVA results and pairwise comparisons for outcomes (all mental health issues, participants *without* prior help-seeking experience only).**

| Subsample *without* prior help-seeking | | | | | | | |
| --- | --- | --- | --- | --- | --- | --- | --- |
|  | total  *N*=770 | CG  *n*=308 | INT1  *n*=226 | INT2  *n*=236 | *F* (2,762) | *p* | Pairwise comparisons |
| Potential help-seeking (GHSQ)^a^ |  | | | | | | |
| Professional max. *M (SD)* | 4.28 (1.79) | 4.20 (1.86) | 4.39 (1.72) | 4.28 (1.77) | .88 | .415 |  |
| Informal max. *M (SD)* | 5.96 (1.31) | 5.99 (1.25) | 5.88 (1.35) | 6.00 (1.35) | 1.17 | .312 |  |
| None *M (SD)* | 3.13 (1.97) | 3.13 (1.97) | 3.30 (1.95) | 2.96 (1.97) | 2.62 | .074 |  |
| Stigma (USS)^b^ |  | | | | | | |
| Blame *M (SD)* | 4.39 (.69) | 4.31 (.75) | 4.44 (.71) | 4.45 (.57) | 3.21 | .041 | INT2 > CG; trend for INT1 > CG (*p*=.054) |
| Distrust *M (SD)* | 3.86 (.78) | 3.77 (.80) | 3.88 (.78) | 3.95 (.76) | 3.74 | .024 | INT2 > CG |
| Help-seeking attitudes (IASMHS)^b^ |  | | | | | | |
| Psychological Openness *M (SD)* | 20.66 (4.91) | 20.56 (4.79) | 21.04 (4.95) | 20.44 (5.04) | .40 | .672 |  |
| Help-seeking propensity *M (SD)* | 19.78 (4.91) | 19.65 (5.15) | 19.85 (4.53) | 19.89 (4.96) | .30 | .739 |  |
| Indifference to stigma *M (SD)* | 23.31 (5.97) | 23.99 (5.63) | 22.98 (6.20) | 22.75 (6.12) | 3.74 | .024 | CG > INT1, INT2 |
| Video acceptability and transportation^a^ | | | | | | | |
| General likability *M (SD)* | 3.98 (.82) | 3.88 (.79) | 4.13 (.80) | 3.96 (.85) | 6.31 | .002 | INT1 > CG, INT2 |
| Comprehensibility *M (SD)* | 4.81 (.47) | 4.79 (.52) | 4.79 (.49) | 4.86 (.37) | 2.07 | .126 |  |
| Interestingness *M (SD)* | 3.87 (.96) | 3.85 (.94) | 3.99 (.92) | 3.79 (1.01) | 2.65 | .071 |  |
| Transportation (TS-SF) *M (SD)* | 4.41 (1.20) | 4.46 (1.16) | 4.47 (1.22) | 4.30 (1.22) | 1.96 | .142 |  |

*Note.* *CG* = control group; *GHSQ* = General Help Seeking Questionnaire; *IASMHS* = Inventory of Attitudes toward Seeking Mental Health Services; *INT1* = intervention 1; *INT2* = intervention 2; *TS-SF* = Transportation Scale - Short Form; *USS* = Universal Stigma Scale. ^a^ Higher scores represent a greater level of agreement. ^b^ Higher scores represent more positive attitudes towards mental health issues and help-seeking. Results controlled for MH issue (random factor) and age (covariate).
