# Supplementary material for: The Impact of Video-Based Microinterventions on Attitudes Toward Mental Health and Help Seeking in Youth: Web-Based Randomized Controlled Trial
Source: J Med Internet Res. 2024 Apr 24;26:e54478. doi: 10.2196/54478 (PMC11079770; doi:10.2196/54478)
Supplement: Multimedia Appendix 2 [file jmir_v26i1e54478_app2.doc]

**Multimedia Appendix**

# **Appendix 2: Separate ANCOVA results and pairwise comparisons for outcomes per mental health problem.**

## **Table S1. ANCOVA results and pairwise comparisons for outcomes (Generalized Anxiety MH issue** conditions only).

| GAD sample | | | | | | | |
| --- | --- | --- | --- | --- | --- | --- | --- |
|  | total  *N*=294 | CG  *n*=111 | INT1  *n*=92 | INT2  *n*=91 | *F* (2,288) | *p* | Pairwise comparisons |
| Potential help-seeking (GHSQ)^a^ |  | | | | | | |
| Professional max. *M (SD)* | 4.39 (1.90) | 4.49 (1.91) | 4.43 (1.85) | 4.24 (1.93) | .35 | .708 |  |
| Informal max. *M (SD)* | 5.93 (1.33) | 6.09 (1.13) | 5.75 (1.43) | 5.92 (1.42) | 2.64 | .073 |  |
| None *M (SD)* | 3.20 (2.08) | 2.99 (1.93) | 3.33 (2.16) | 3.34 (2.17) | 1.50 | .226 |  |
| Stigma (USS)^b^ |  | | | | | | |
| Blame *M (SD)* | 4.55 (.61) | 4.54 (.63) | 4.62 (.54) | 4.50 (.66) | .87 | .421 |  |
| Distrust *M (SD)* | 4.10 (.69) | 4.06 (.66) | 4.18 (.67) | 4.08 (.76) | 1.28 | .281 |  |
| Help-seeking attitudes (IASMHS)^b^ |  | | | | | | |
| Psychological Openness *M (SD)* | 20.93 (4.93) | 20.86 (5.13) | 21.27 (4.59) | 20.68 (5.06) | .17 | .846 |  |
| Help-seeking propensity *M (SD)* | 20.47 (5.67) | 20.11 (5.73) | 20.75 (5.27) | 20.63 (6.00) | .44 | .644 |  |
| Indifference to stigma *M (SD)* | 23.17 (6.61) | 24.25 (6.07) | 22.53 (6.47) | 22.49 (7.26) | 3.22 | .041 | CG > INT1; trend for CG > INT2 (*p*=.056) |
| Video acceptability and transportation^a^ | | | | | | | |
| General likability *M (SD)* | 4.04 (.74) | 3.90 (.71) | 4.26 (.71) | 3.98 (.75) | 7.63 | <.001 | INT1 > CG, INT2 |
| Comprehensiveness *M (SD)* | 4.86 (.36) | 4.87 (.33) | 4.85 (.39) | 4.86 (.35) | .08 | .923 |  |
| Interestingness *M (SD)* | 4.02 (.87) | 3.97 (.80) | 4.29 (.86) | 3.80 (.89) | 9.02 | <.001 | INT1 > CG, INT2 |
| Transportation (TS-SF) *M (SD)* | 4.60 (1.20) | 4.56 (1.15) | 4.68 (1.19) | 4.57 (1.28) | .82 | .44 |  |

*Note.* *CG* = control group; *GHSQ* = General Help Seeking Questionnaire; *IASMHS* = Inventory of Attitudes toward Seeking Mental Health Services; *INT1* = intervention 1; *INT2* = intervention 2; *TS-SF* = Transportation Scale - Short Form*; USS* = Universal Stigma Scale. ^a^ Higher scores represent a greater level of agreement. ^b^ Higher scores represent more positive attitudes towards mental health issues and help-seeking. Results controlled for help-seeking (fixed factor), MH issue (random factor), age, and GAD-7 score (covariates).

## **Table S2. ANCOVA results and pairwise comparisons for outcomes** (depression conditions only).

| Depression sample | | | | | | | |
| --- | --- | --- | --- | --- | --- | --- | --- |
|  | total  *N*=261 | CG  *n*=111 | INT1  *n*=72 | INT2  *n*=78 | *F* (2,255) | *p* | Pairwise comparisons |
| Potential help-seeking (GHSQ)^a^ |  | | | | | | |
| Professional max. *M (SD)* | 4.51 (1.91) | 4.32 (1.89) | 4.56 (1.99) | 4.74 (1.87) | 1.64 | .195 |  |
| Informal max. *M (SD)* | 5.90 (1.39) | 5.79 (1.45) | 6.07 (1.23) | 5.90 (1.44) | .86 | .426 |  |
| None *M (SD)* | 3.33 (2.10) | 3.52 (2.23) | 3.29 (1.94) | 3.09 (2.06) | .83 | .438 |  |
| Stigma (USS)^b^ |  | | | | | | |
| Blame *M (SD)* | 4.67 (.54) | 4.61 (.63) | 4.70 (.50) | 4.71 (.41) | 1.04 | .354 |  |
| Distrust *M (SD)* | 3.93 (.73) | 3.86 (.68) | 3.94 (.79) | 4.03 (.72) | 1.51 | .223 |  |
| Help-seeking attitudes (IASMHS)^b^ |  | | | | | | |
| Psychological Openness *M (SD)* | 20.80 (4.74) | 20.68 (4.58) | 22.10 (4.79) | 19.77 (4.71) | 4.59 | .011 | INT1 > CG, INT2 |
| Help-seeking propensity *M (SD)* | 20.68 (5.50) | 20.70 (5.66) | 20.78 (5.35) | 20.56 (5.45) | .03 | .970 |  |
| Indifference to stigma *M (SD)* | 22.59 (7.01) | 22.57 (6.92) | 22.75 (7.56) | 22.46 (6.68) | .03 | .967 |  |
| Video acceptability and transportation^a^ | | | | | | | |
| General likability *M (SD)* | 3.98 (.81) | 3.98 (.79) | 4.07 (.76) | 3.88 (.90) | 1.00 | .370 |  |
| Comprehensiveness *M (SD)* | 4.81 (.48) | 4.75 (.58) | 4.86 (.39) | 4.86 (.39) | 1.79 | .169 |  |
| Interestingness *M (SD)* | 3.84 (.98) | 3.86 (.89) | 3.89 (1.04) | 3.77 (1.04) | .31 | .736 |  |
| Transportation (TS-SF) *M (SD)* | 4.90 (1.21) | 5.01 (1.19) | 4.79 (1.29) | 4.83 (1.17) | .86 | .424 |  |

*Note.* *CG* = control group; *GHSQ* = General Help Seeking Questionnaire; *IASMHS* = Inventory of Attitudes toward Seeking Mental Health Services; *INT1* = intervention 1; *INT2* = intervention 2; *TS-SF* = Transportation Scale - Short Form*; USS* = Universal Stigma Scale. ^a^ Higher scores represent a greater level of agreement. ^b^ Higher scores represent more positive attitudes towards mental health issues and help-seeking. Results controlled for help-seeking (fixed factor), MH issue (random factor), age, and PHQ-9 score (covariates).

## **Table S3. ANCOVA results and pairwise comparisons for outcomes** (bulimia conditions only).

| Bulimia sample | | | | | | | |
| --- | --- | --- | --- | --- | --- | --- | --- |
|  | total  *N*=277 | CG  *n*=108 | INT1  *n*=81 | INT2  *n*=88 | *F* (2,271) | *p* | Pairwise comparisons |
| Potential help-seeking (GHSQ)^a^ |  | | | | | | |
| Professional max. *M (SD)* | 4.83 (1.74) | 4.62 (1.90) | 5.04 (1.65) | 4.89 (1.61) | 1.16 | .316 |  |
| Informal max. *M (SD)* | 5.80 (1.43) | 5.67 (1.58) | 5.84 (1.29) | 5.93 (1.35) | .92 | .399 |  |
| None *M (SD)* | 2.96 (2.00) | 3.34 (2.11) | 2.69 (1.86) | 2.75 (1.94) | 3.04 | .050 | CG > INT1; trend for CG > INT2 (.051) |
| Stigma (USS)^b^ |  | | | | | | |
| Blame *M (SD)* | 4.50 (.62) | 4.41 (.63) | 4.59 (.66) | 4.53 (.56) | 1.67 | .191 |  |
| Distrust *M (SD)* | 4.30 (.61) | 4.24 (.59) | 4.35 (.64) | 4.33 (.60) | .74 | .479 |  |
| Help-seeking attitudes (IASMHS)^b^ |  | | | | | | |
| Psychological Openness *M (SD)* | 21.82 (5.00) | 21.54 (4.90) | 22.49 (4.66) | 21.55 (5.41) | 1.06 | .348 |  |
| Help-seeking propensity *M (SD)* | 21.30 (4.96) | 20.31 (4.99) | 22.01 (5.01) | 21.88 (4.72) | 3.27 | .040 | INT1, INT2 > CG |
| Indifference to stigma *M (SD)* | 23.99 (5.90) | 24.04 (5.70) | 25.06 (5.32) | 22.95 (6.50) | 3.45 | .033 | INT1 > INT2 |
| Video acceptability and transportation^a^ | | | | | | | |
| General likability *M (SD)* | 3.86 (.84) | 3.79 (.77) | 4.01 (.86) | 3.82 (.89) | 1.81 | .165 |  |
| Comprehensiveness *M (SD)* | 4.84 (.38) | 4.87 (.34) | 4.77 (.46) | 4.89 (.35) | 2.54 | .081 |  |
| Interestingness *M (SD)* | 3.81 (1.02) | 3.89 (.96) | 3.99 (.99) | 3.55 (1.06) | 4.49 | .012 | CG, INT1 > INT2 |
| Transportation (TS-SF) *M (SD)* | 4.41 (1.27) | 4.51 (1.19) | 4.56 (1.33) | 4.15 (1.28) | 2.49 | .085 |  |

*Note.* *CG* = control group; *GHSQ* = General Help Seeking Questionnaire; *IASMHS* = Inventory of Attitudes toward Seeking Mental Health Services; *INT1* = intervention 1; *INT2* = intervention 2; *TS-SF* = Transportation Scale - Short Form*; USS* = Universal Stigma Scale. ^a^ Higher scores represent a greater level of agreement. ^b^ Higher scores represent more positive attitudes towards mental health issues and help-seeking. Results controlled for help-seeking (fixed factor), MH issue (random factor), age, and WCS score (covariates).

## **Table S4. ANCOVA results and pairwise comparisons for outcomes** (NSSI conditions only).

| NSSI sample | | | | | | | |
| --- | --- | --- | --- | --- | --- | --- | --- |
|  | total  *N*=283 | CG  *n*=112 | INT1  *n*=81 | INT2  *n*=90 | *F* (2,277) | *p* | Pairwise comparisons |
| Potential help-seeking (GHSQ)^a^ |  | | | | | | |
| Professional max. *M (SD)* | 4.82 (1.71) | 4.78 (1.82) | 4.88 (1.50) | 4.82 (1.74) | .13 | .878 |  |
| Informal max. *M (SD)* | 5.60 (1.51) | 5.63 (1.50) | 5.27 (1.60) | 5.84 (1.41) | 2.68 | .071 |  |
| None *M (SD)* | 3.27 (2.04) | 3.16 (1.98) | 3.77 (2.01) | 2.96 (2.07) | 2.78 | .064 |  |
| Stigma (USS)^b^ |  | | | | | | |
| Blame *M (SD)* | 4.54 (.56) | 4.51 (.59) | 4.51 (.56) | 4.60 (.52) | .69 | .502 |  |
| Distrust *M (SD)* | 4.12 (.68) | 4.00 (.67) | 4.16 (.62) | 4.24 (.73) | 2.90 | .057 | INT2 > CG |
| Help-seeking attitudes (IASMHS)^b^ |  | | | | | | |
| Psychological Openness *M (SD)* | 21.07 (4.69) | 20.81 (4.81) | 20.90 (4.78) | 21.56 (4.46) | .64 | .526 |  |
| Help-seeking propensity *M (SD)* | 20.50 (5.35) | 20.82 (5.79) | 19.65 (4.54) | 20.87 (5.41) | 1.23 | .294 |  |
| Indifference to stigma *M (SD)* | 22.98 (6.19) | 23.41 (6.00) | 21.54 (6.90) | 23.74 (5.58) | 2.82 | .062 |  |
| Video acceptability and transportation^a^ | | | | | | | |
| General likability *M (SD)* | 4.02 (.80) | 3.79 (.83) | 4.30 (.62) | 4.07 (.83) | 10.31 | <.001 | INT1, INT2 > CG |
| Comprehensiveness *M (SD)* | 4.81 (.46) | 4.77 (.50) | 4.84 (.40) | 4.83 (.46) | .71 | .493 |  |
| Interestingness *M (SD)* | 4.01 (.91) | 3.87 (.95) | 4.10 (.77) | 4.11 (.94) | 2.36 | .096 |  |
| Transportation (TS-SF) *M (SD)* | 4.68 (1.16) | 4.60 (1.21) | 4.92 (1.00) | 4.57 (1.20) | 2.17 | .117 |  |

*Note.* *CG* = control group; *GHSQ* = General Help Seeking Questionnaire; *IASMHS* = Inventory of Attitudes toward Seeking Mental Health Services; *INT1* = intervention 1; *INT2* = intervention 2; *TS-SF* = Transportation Scale - Short Form*; USS* = Universal Stigma Scale. ^a^ Higher scores represent a greater level of agreement. ^b^ Higher scores represent more positive attitudes towards mental health issues and help-seeking. Results controlled for help-seeking (fixed factor), MH issue (random factor), age, and number of NSSI events during the past 12 months (SITBI-G) (covariates).

## **Table S5. ANCOVA results and pairwise comparisons for outcomes (problematic alcohol use** conditions only).

| Problematic alcohol use sample | | | | | | | |
| --- | --- | --- | --- | --- | --- | --- | --- |
|  | total  *N*=279 | CG  *n*=112 | INT1  *n*=84 | INT2  *n*=83 | *F* (2,273) | *p* | Pairwise comparisons |
| Potential help-seeking (GHSQ)^a^ |  | | | | | | |
| Professional max. *M (SD)* | 5.13 (1.71) | 5.05 (1.81) | 5.21 (1.59) | 5.14 (1.71) | .10 | .905 |  |
| Informal max. *M (SD)* | 6.05 (1.21) | 6.18 (1.04) | 5.75 (1.46) | 6.17 (1.10) | 3.51 | .031 | CG, INT2 > INT1 |
| None *M (SD)* | 2.60 (1.75) | 2.39 (1.68) | 2.93 (1.82) | 2.54 (1.73) | 2.48 | .086 |  |
| Stigma (USS)^b^ |  | | | | | | |
| Blame *M (SD)* | 4.08 (.72) | 4.00 (.75) | 4.11 (.79) | 4.16 (.59) | 1.12 | .329 |  |
| Distrust *M (SD)* | 3.21 (.74) | 3.07 (.76) | 3.22 (.71) | 3.39 (.72) | 4.49 | .012 | INT2 > CG |
| Help-seeking attitudes (IASMHS)^b^ |  | | | | | | |
| Psychological Openness *M (SD)* | 21.37 (4.69) | 21.39 (4.80) | 21.57 (4.67) | 21.14 (4.63) | .086 | .918 |  |
| Help-seeking propensity *M (SD)* | 21.82 (4.81) | 21.92 (4.68) | 22.00 (5.23) | 21.49 (4.60) | .22 | .801 |  |
| Indifference to stigma *M (SD)* | 24.11 (5.67) | 24.87 (5.34) | 23.25 (5.85) | 23.95 (5.84) | 2.41 | .092 |  |
| Video acceptability and transportation^a^ | | | | | | | |
| General likability *M (SD)* | 3.78 (.82) | 3.79 (.79) | 3.82 (.88) | 3.73 (.81) | .27 | .762 |  |
| Comprehensiveness *M (SD)* | 4.75 (.56) | 4.69 (.63) | 4.77 (.57) | 4.81 (.45) | 1.12 | .326 |  |
| Interestingness *M (SD)* | 3.60 (.96) | 3.63 (1.02) | 3.61 (.92) | 3.54 (.93) | .24 | .787 |  |
| Transportation (TS-SF) *M (SD)* | 3.89 (1.15) | 3.97 (1.11) | 3.96 (1.21) | 3.72 (1.12) | 1.54 | .217 |  |

*Note.* *CG* = control group; *GHSQ* = General Help Seeking Questionnaire; *IASMHS* = Inventory of Attitudes toward Seeking Mental Health Services; *INT1* = intervention 1; *INT2* = intervention 2; *TS-SF* = Transportation Scale - Short Form*; USS* = Universal Stigma Scale. ^a^ Higher scores represent a greater level of agreement. ^b^ Higher scores represent more positive attitudes towards mental health issues and help-seeking. Results controlled for help-seeking (fixed factor), MH issue (random factor), age, and AUDIT-C score (covariates).
