# Supplementary material for: The Impact of Video-Based Microinterventions on Attitudes Toward Mental Health and Help Seeking in Youth: Web-Based Randomized Controlled Trial
Source: J Med Internet Res. 2024 Apr 24;26:e54478. doi: 10.2196/54478 (PMC11079770; doi:10.2196/54478)
Supplement: Multimedia Appendix 3 [file jmir_v26i1e54478_app3.doc]

**Multimedia Appendix**

# **Appendix 3: Separate ANCOVA results and pairwise comparisons for participants *with* and *without* prior help-seeking experience per mental health problem.**

## **Table S1. ANCOVA results and pairwise comparisons for outcomes (GAD-sample, participants *with*** prior help-seeking experience only).

| GAD subsample *with* prior help-seeking | | | | | | | |
| --- | --- | --- | --- | --- | --- | --- | --- |
|  | total  *N*=113 | CG  *n*=45 | INT1  *n*=34 | INT2  *n*=34 | *F* (2,108) | *p* | Pairwise comparisons |
| Potential help-seeking (GHSQ)^a^ |  | | | | | | |
| Professional max. *M (SD)* | 5.21 (1.68) | 5.27 (1.75) | 5.12 (1.63) | 5.24 (1.69) | .08 | .926 |  |
| Informal max. *M (SD)* | 5.88 (1.36) | 6.04 (1.21) | 5.41 (1.48) | 6.12 (1.37) | 2.86 | .062 |  |
| None *M (SD)* | 2.94 (2.21) | 2.58 (1.99) | 3.35 (2.41) | 3.00 (2.24) | 1.46 | .237 |  |
| Stigma (USS)^b^ |  | | | | | | |
| Blame *M (SD)* | 4.64 (.57) | 4.62 (.59) | 4.66 (.50) | 4.64 (.62) | .06 | .944 |  |
| Distrust *M (SD)* | 4.18 (.69) | 4.07 (.65) | 4.24 (.68) | 4.27 (.74) | 1.05 | .355 |  |
| Help-seeking attitudes (IASMHS)^b^ |  | | | | | | |
| Psychological Openness *M (SD)* | 21.32 (4.62) | 21.18 (5.26) | 21.94 (4.12) | 20.88 (4.25) | .47 | .626 |  |
| Help-seeking propensity *M (SD)* | 22.36 (5.60) | 21.76 (5.63) | 22.21 (5.73) | 23.32 (5.46) | .64 | .531 |  |
| Indifference to stigma *M (SD)* | 22.98 (6.92) | 23.29 (6.19) | 21.62 (7.53) | 23.94 (7.19) | .85 | .429 |  |
| Video acceptability and transportation^a^ | | | | | | | |
| General likability *M (SD)* | 4.01 (.75) | 3.89 (.78) | 4.24 (.70) | 3.94 (.74) | 2.37 | .098 |  |
| Comprehensiveness *M (SD)* | 4.86 (.35) | 4.89 (.32) | 4.85 (.36) | 4.82 (.39) | .25 | .782 |  |
| Interestingness *M (SD)* | 4.08 (.85) | 4.09 (.76) | 4.29 (.91) | 3.85 (.86) | 2.24 | .112 |  |
| Transportation (TS-SF) *M (SD)* | 4.76 (1.23) | 4.70 (1.12) | 4.91 (1.19) | 4.68 (1.42) | .32 | .730 |  |

*Note.* *CG* = control group; *GHSQ* = General Help Seeking Questionnaire; *IASMHS* = Inventory of Attitudes toward Seeking Mental Health Services; *INT1* = intervention 1; *INT2* = intervention 2; *TS-SF* = Transportation Scale - Short Form*; USS* = Universal Stigma Scale. ^a^ Higher scores represent a greater level of agreement. ^b^ Higher scores represent more positive attitudes towards mental health issues and help-seeking. Results controlled for MH issue (random factor), age, and GAD-7 score (covariates).

## **Table S2. ANCOVA results and pairwise comparisons for outcomes** (GAD-sample, participants without prior help-seeking experience only).

| GAD subsample *without* prior help-seeking | | | | | | | |
| --- | --- | --- | --- | --- | --- | --- | --- |
|  | total  *N*=181 | CG  *n*=66 | INT1  *n*=58 | INT2  *n*=57 | *F* (2,176) | *p* | Pairwise comparisons |
| Potential help-seeking (GHSQ)^a^ |  | | | | | | |
| Professional max. *M (SD)* | 3.88 (1.85) | 3.95 (1.84) | 4.03 (1.86) | 3.65 (1.84) | .45 | .639 |  |
| Informal max. *M (SD)* | 5.97 (1.30) | 6.12 (1.07) | 5.95 (1.38) | 5.81 (1.46) | 1.07 | .346 |  |
| None *M (SD)* | 3.37 (1.99) | 3.27 (1.85) | 3.31 (2.03) | 3.54 (2.12) | .30 | .740 |  |
| Stigma (USS)^b^ |  | | | | | | |
| Blame *M (SD)* | 4.50 (.63) | 4.49 (.65) | 4.59 (.57) | 4.42 (.68) | 1.01 | .365 |  |
| Distrust *M (SD)* | 4.06 (.70) | 4.05 (.67) | 4.16 (.68) | 3.96 (.75) | 1.43 | .241 |  |
| Help-seeking attitudes (IASMHS)^b^ |  | | | | | | |
| Psychological Openness *M (SD)* | 20.69 (5.11) | 20.64 (5.06) | 20.88 (4.83) | 20.56 (5.52) | .002 | .998 |  |
| Help-seeking propensity *M (SD)* | 19.29 (5.39) | 18.98 (5.57) | 19.90 (4.82) | 19.02 (5.76) | .12 | .890 |  |
| Indifference to stigma *M (SD)* | 23.29 (6.43) | 24.91 (5.94) | 23.07 (5.76) | 21.63 (7.22) | 4.48 | .013 | CG > INT1, INT2 |
| Video acceptability and transportation^a^ | | | | | | | |
| General likability *M (SD)* | 4.06 (.73) | 3.91 (.67) | 4.28 (.72) | 4.00 (.76) | 4.60 | .011 | INT1 > CG, INT2 |
| Comprehensiveness *M (SD)* | 4.86 (.36) | 4.86 (.35) | 4.84 (.41) | 4.88 (.33) | .050 | .951 |  |
| Interestingness *M (SD)* | 3.98 (.88) | 3.89 (.83) | 4.29 (.84) | 3.77 (.91) | 6.17 | .003 | INT1 > CG, INT2 |
| Transportation (TS-SF) *M (SD)* | 4.50 (1.17) | 4.47 (1.16) | 4.54 (1.17) | 4.50 (1.20) | .34 | .716 |  |

*Note.* *CG* = control group; *GHSQ* = General Help Seeking Questionnaire; *IASMHS* = Inventory of Attitudes toward Seeking Mental Health Services; *INT1* = intervention 1; *INT2* = intervention 2; *TS-SF* = Transportation Scale - Short Form*; USS* = Universal Stigma Scale. ^a^ Higher scores represent a greater level of agreement. ^b^ Higher scores represent more positive attitudes towards mental health issues and help-seeking. Results controlled for MH issue (random factor), age, and GAD-7 score (covariates).

## **Table S3. ANCOVA results and pairwise comparisons for outcomes** (Depression-sample, participants with prior help-seeking experience only).

| Depression subsample *with* prior help-seeking | | | | | | | |
| --- | --- | --- | --- | --- | --- | --- | --- |
|  | total  *N*=118 | CG  *n*=54 | INT1  *n*=32 | INT2  *n*=32 | *F* (2,113) | *p* | Pairwise comparisons |
| Potential help-seeking (GHSQ)^a^ |  | | | | | | |
| Professional max. *M (SD)* | 5.04 (1.83) | 4.80 (1.91) | 4.75 (2.02) | 5.75 (1.27) | 2.92 | .058 | INT2 > CG, INT1 |
| Informal max. *M (SD)* | 5.83 (1.42) | 5.63 (1.66) | 5.97 (1.20) | 6.03 (1.12) | .98 | .377 |  |
| None *M (SD)* | 3.43 (2.18) | 3.57 (2.36) | 3.19 (1.93) | 3.44 (2.14) | .56 | .572 |  |
| Stigma (USS)^b^ |  | | | | | | |
| Blame *M (SD)* | 4.70 (.46) | 4.69 (.48) | 4.68 (.47) | 4.75 (.43) | .23 | .794 |  |
| Distrust *M (SD)* | 4.00 (.73) | 3.90 (.72) | 3.97 (.76) | 4.20 (.72) | 1.42 | .246 |  |
| Help-seeking attitudes (IASMHS)^b^ |  | | | | | | |
| Psychological Openness *M (SD)* | 21.51 (5.00) | 21.24 (5.27) | 22.47 (4.98) | 21.00 (4.53) | 1.29 | .279 |  |
| Help-seeking propensity *M (SD)* | 21.99 (5.86) | 22.07 (6.13) | 21.47 (5.95) | 22.38 (5.44) | .05 | .948 |  |
| Indifference to stigma *M (SD)* | 23.22 (7.57) | 23.09 (7.23) | 22.53 (8.21) | 24.13 (7.63) | .09 | .915 |  |
| Video acceptability and transportation^a^ | | | | | | | |
| General likability *M (SD)* | 3.93 (.81) | 4.02 (.77) | 4.00 (.67) | 3.72 (.99) | 1.51 | .225 |  |
| Comprehensiveness *M (SD)* | 4.84 (.39) | 4.76 (.47) | 4.91 (.30) | 4.91 (.30) | 2.14 | .123 |  |
| Interestingness *M (SD)* | 3.81 (.95) | 3.83 (.86) | 3.84 (1.02) | 3.72 (1.05) | .18 | .832 |  |
| Transportation (TS-SF) *M (SD)* | 4.99 (1.27) | 5.06 (1.28) | 4.85 (1.32) | 5.01 (1.22) | .36 | .697 |  |

*Note.* *CG* = control group; *GHSQ* = General Help Seeking Questionnaire; *IASMHS* = Inventory of Attitudes toward Seeking Mental Health Services; *INT1* = intervention 1; *INT2* = intervention 2; *TS-SF* = Transportation Scale - Short Form*; USS* = Universal Stigma Scale. ^a^ Higher scores represent a greater level of agreement. ^b^ Higher scores represent more positive attitudes towards mental health issues and help-seeking. Results controlled for MH issue (random factor), age, and PHQ-9 score (covariates).

## **Table S4. ANCOVA results and pairwise comparisons for outcomes** (Depression-sample, participants without prior help-seeking experience only).

| Depression subsample *without* prior help-seeking | | | | | | | |
| --- | --- | --- | --- | --- | --- | --- | --- |
|  | total  *N*=143 | CG  *n*=57 | INT1  *n*=40 | INT2  *n*=46 | *F* (2,138) | *p* | Pairwise comparisons |
| Potential help-seeking (GHSQ)^a^ |  | | | | | | |
| Professional max. *M (SD)* | 4.07 (1.87) | 3.86 (1.77) | 4.40 (1.97) | 4.04 (1.91) | .86 | .426 |  |
| Informal max. *M (SD)* | 5.96 (1.36) | 5.95 (1.20) | 6.15 (1.25) | 5.80 (1.63) | .66 | .518 |  |
| None *M (SD)* | 3.24 (2.04) | 3.47 (2.12) | 3.38 (1.97) | 2.85 (1.99) | 1.36 | .261 |  |
| Stigma (USS)^b^ |  | | | | | | |
| Blame *M (SD)* | 4.64 (.59) | 4.54 (.74) | 4.72 (.53) | 4.68 (.40) | 1.27 | .283 |  |
| Distrust *M (SD)* | 3.88 (.72) | 3.82 (.65) | 3.92 (.82) | 3.92 (.71) | .42 | .660 |  |
| Help-seeking attitudes (IASMHS)^b^ |  | | | | | | |
| Psychological Openness *M (SD)* | 20.22 (4.46) | 20.16 (3.77) | 21.80 (4.69) | 18.91 (4.69) | 4.20 | .017 | INT1 > INT2 |
| Help-seeking propensity *M (SD)* | 19.60 (4.94) | 19.40 (4.89) | 20.23 (4.83) | 19.30 (5.15) | .28 | .758 |  |
| Indifference to stigma *M (SD)* | 22.06 (6.49) | 22.07 (6.65) | 22.93 (7.10) | 21.30 (5.73) | .57 | .566 |  |
| Video acceptability and transportation^a^ | | | | | | | |
| General likability *M (SD)* | 4.01 (.81) | 3.95 (.81) | 4.13 (.82) | 4.00 (.82) | .66 | .521 |  |
| Comprehensiveness *M (SD)* | 4.79 (.54) | 4.74 (.67) | 4.83 (.45) | 4.83 (.44) | .46 | .633 |  |
| Interestingness *M (SD)* | 3.87 (1.00) | 3.88 (.93) | 3.93 (1.07) | 3.80 (1.05) | .14 | .871 |  |
| Transportation (TS-SF) *M (SD)* | 4.82 (1.16) | 4.97 (1.10) | 4.74 (1.28) | 4.70 (1.14) | .67 | .514 |  |

*Note.* *CG* = control group; *GHSQ* = General Help Seeking Questionnaire; *IASMHS* = Inventory of Attitudes toward Seeking Mental Health Services; *INT1* = intervention 1; *INT2* = intervention 2; *TS-SF* = Transportation Scale - Short Form*; USS* = Universal Stigma Scale. ^a^ Higher scores represent a greater level of agreement. ^b^ Higher scores represent more positive attitudes towards mental health issues and help-seeking. Results controlled for MH issue (random factor), age, and PHQ-9 score (covariates).

## **Table S5. ANCOVA results and pairwise comparisons for outcomes (bulimia** sample, participants with prior help-seeking experience only).

| Bulimia subsample *with* prior help-seeking | | | | | | | |
| --- | --- | --- | --- | --- | --- | --- | --- |
|  | total  *N*=145 | CG  *n*=54 | INT1  *n*=43 | INT2  *n*=48 | *F* (2,140) | *p* | Pairwise comparisons |
| Potential help-seeking (GHSQ)^a^ |  | | | | | | |
| Professional max. *M (SD)* | 5.26 (1.71) | 5.06 (1.83) | 5.56 (1.53) | 5.23 (1.72) | .99 | .374 |  |
| Informal max. *M (SD)* | 5.61 (1.60) | 5.30 (1.82) | 5.81 (1.35) | 5.77 (1.51) | 1.63 | .201 |  |
| None *M (SD)* | 2.76 (2.06) | 3.04 (2.13) | 2.56 (1.98) | 2.63 (2.07) | .79 | .455 |  |
| Stigma (USS)^b^ |  | | | | | | |
| Blame *M (SD)* | 4.61 (.57) | 4.53 (.57) | 4.67 (.57) | 4.63 (.59) | .76 | .469 |  |
| Distrust *M (SD)* | 4.44 (.56) | 4.35 (.51) | 4.53 (.61) | 4.45 (.56) | 1.25 | .291 |  |
| Help-seeking attitudes (IASMHS)^b^ |  | | | | | | |
| Psychological Openness *M (SD)* | 22.63 (4.74) | 21.91 (4.75) | 23.16 (4.21) | 22.96 (5.16) | 1.00 | .369 |  |
| Help-seeking propensity *M (SD)* | 22.69 (5.20) | 21.72 (5.01) | 23.14 (5.46) | 23.38 (5.13) | 1.46 | .237 |  |
| Indifference to stigma *M (SD)* | 23.94 (6.44) | 23.94 (6.28) | 24.95 (5.90) | 23.02 (7.06) | 1.81 | .167 |  |
| Video acceptability and transportation^a^ | | | | | | | |
| General likability *M (SD)* | 3.81 (.81) | 3.70 (.74) | 3.95 (.90) | 3.79 (.80) | 1.06 | .349 |  |
| Comprehensiveness *M (SD)* | 4.84 (.39) | 4.87 (.34) | 4.74 (.49) | 4.90 (.31) | 2.00 | .139 |  |
| Interestingness *M (SD)* | 3.76 (1.00) | 3.76 (1.03) | 3.95 (.98) | 3.58 (.96) | 1.47 | .235 |  |
| Transportation (TS-SF) *M (SD)* | 4.52 (1.30) | 4.50 (1.19) | 4.87 (1.34) | 4.23 (1.32) | 1.76 | .176 |  |

*Note.* *CG* = control group; *GHSQ* = General Help Seeking Questionnaire; *IASMHS* = Inventory of Attitudes toward Seeking Mental Health Services; *INT1* = intervention 1; *INT2* = intervention 2; *TS-SF* = Transportation Scale - Short Form*; USS* = Universal Stigma Scale. ^a^ Higher scores represent a greater level of agreement. ^b^ Higher scores represent more positive attitudes towards mental health issues and help-seeking. Results controlled for MH issue (random factor), age, and PHQ-9 score (covariates).

## **Table S6. ANCOVA results and pairwise comparisons for outcomes (bulimia** sample, participants without prior help-seeking experience only).

| Bulimia subsample *without* prior help-seeking | | | | | | | |
| --- | --- | --- | --- | --- | --- | --- | --- |
|  | total  *N*=132 | CG  *n*=54 | INT1  *n*=38 | INT2  *n*=40 | *F* (2,127) | *p* | Pairwise comparisons |
| Potential help-seeking (GHSQ)^a^ |  | | | | | | |
| Professional max. *M (SD)* | 4.35 (1.66) | 4.19 (1.88) | 4.45 (1.61) | 4.47 (1.38) | .52 | .596 |  |
| Informal max. *M (SD)* | 6.02 (1.19) | 6.04 (1.21) | 5.87 (1.23) | 6.13 (1.11) | .74 | .478 |  |
| None *M (SD)* | 3.19 (1.91) | 3.65 (2.06) | 2.84 (1.72) | 2.90 (1.78) | 2.95 | .056 | CG > INT2; trend for CG > INT1 (*p*=.059) |
| Stigma (USS)^b^ |  | | | | | | |
| Blame *M (SD)* | 4.38 (.64) | 4.29 (.66) | 4.48 (.74) | 4.41 (.50) | .87 | .423 |  |
| Distrust *M (SD)* | 4.15 (.63) | 4.12 (.65) | 4.14 (.61) | 4.18 (.64) | .13 | .875 |  |
| Help-seeking attitudes (IASMHS)^b^ |  | | | | | | |
| Psychological Openness *M (SD)* | 20.93 (5.15) | 21.17 (5.07) | 21.74 (5.07) | 19.85 (5.27) | 1.06 | .350 |  |
| Help-seeking propensity *M (SD)* | 19.78 (4.19) | 18.89 (4.58) | 20.74 (4.14) | 20.08 (3.47) | 2.18 | .117 |  |
| Indifference to stigma *M (SD)* | 24.05 (5.26) | 24.13 (5.11) | 25.18 (4.64) | 22.88 (5.85) | 1.44 | .240 |  |
| Video acceptability and transportation^a^ | | | | | | | |
| General likability *M (SD)* | 3.92 (.87) | 3.87 (.80) | 4.08 (.82) | 3.85 (1.00) | .80 | .452 |  |
| Comprehensiveness *M (SD)* | 4.85 (.38) | 4.87 (.34) | 4.79 (.41) | 4.88 (.40) | .65 | .523 |  |
| Interestingness *M (SD)* | 3.86 (1.04) | 4.02 (.88) | 4.03 (1.03) | 3.50 (1.18) | 3.81 | .025 | CG, INT1 > INT2 |
| Transportation (TS-SF) *M (SD)* | 4.29 (1.23) | 4.52 (1.19) | 4.21 (1.26) | 4.05 (1.25) | 2.06 | .132 |  |

*Note.* *CG* = control group; *GHSQ* = General Help Seeking Questionnaire; *IASMHS* = Inventory of Attitudes toward Seeking Mental Health Services; *INT1* = intervention 1; *INT2* = intervention 2; *TS-SF* = Transportation Scale - Short Form*; USS* = Universal Stigma Scale. ^a^ Higher scores represent a greater level of agreement. ^b^ Higher scores represent more positive attitudes towards mental health issues and help-seeking. Results controlled for MH issue (random factor), age, and WCS score (covariates).

## **Table S7. ANCOVA results and pairwise comparisons for outcomes** (NSSI sample, participants with prior help-seeking experience only).

| NSSI subsample *with* prior help-seeking | | | | | | | |
| --- | --- | --- | --- | --- | --- | --- | --- |
|  | total  *N*=118 | CG  *n*=44 | INT1  *n*=34 | INT2  *n*=40 | *F* (2,113) | *p* | Pairwise comparisons |
| Potential help-seeking (GHSQ)^a^ |  | | | | | | |
| Professional max. *M (SD)* | 5.28 (1.60) | 5.36 (1.53) | 5.26 (1.62) | 5.20 (1.68) | .09 | .918 |  |
| Informal max. *M (SD)* | 5.26 (1.59) | 5.45 (1.39) | 4.76 (1.86) | 5.48 (1.50) | 1.58 | .212 |  |
| None *M (SD)* | 3.52 (2.03) | 3.41 (1.95) | 3.94 (2.03) | 3.28 (2.11) | .43 | .650 |  |
| Stigma (USS)^b^ |  | | | | | | |
| Blame *M (SD)* | 4.62 (.48) | 4.60 (.43) | 4.69 (.39) | 4.58 (.58) | .70 | .500 |  |
| Distrust *M (SD)* | 4.26 (.65) | 4.17 (.61) | 4.45 (.53) | 4.21 (.76) | 1.79 | .171 |  |
| Help-seeking attitudes (IASMHS)^b^ |  | | | | | | |
| Psychological Openness *M (SD)* | 21.63 (4.46) | 21.43 (4.65) | 21.88 (4.26) | 21.63 (4.52) | .48 | .619 |  |
| Help-seeking propensity *M (SD)* | 21.54 (5.61) | 21.93 (5.96) | 21.12 (5.09) | 21.48 (5.74) | .05 | .949 |  |
| Indifference to stigma *M (SD)* | 22.22 (7.06) | 22.64 (7.26) | 20.91 (7.53) | 22.88 (6.42) | .26 | .772 |  |
| Video acceptability and transportation^a^ | | | | | | | |
| General likability *M (SD)* | 3.92 (.81) | 3.68 (.83) | 4.29 (.58) | 3.85 (.86) | 6.07 | .003 | INT1 > CG, INT2 |
| Comprehensiveness *M (SD)* | 4.81 (.48) | 4.77 (.48) | 4.91 (.29) | 4.75 (.59) | 1.18 | .310 |  |
| Interestingness *M (SD)* | 4.03 (.91) | 3.91 (.91) | 4.32 (.64) | 3.93 (1.05) | 2.24 | .111 |  |
| Transportation (TS-SF) *M (SD)* | 4.85 (1.22) | 4.89 (1.30) | 5.15 (1.00) | 4.56 (1.25) | 1.51 | .225 |  |

*Note.* *CG* = control group; *GHSQ* = General Help Seeking Questionnaire; *IASMHS* = Inventory of Attitudes toward Seeking Mental Health Services; *INT1* = intervention 1; *INT2* = intervention 2; *TS-SF* = Transportation Scale - Short Form*; USS* = Universal Stigma Scale. ^a^ Higher scores represent a greater level of agreement. ^b^ Higher scores represent more positive attitudes towards mental health issues and help-seeking. Results controlled for MH issue (random factor), age, and number of NSSI events during the past 12 months (SITBI-G) (covariates).

## **Table S8. ANCOVA results and pairwise comparisons for outcomes** (NSSI sample, participants without prior help-seeking experience only).

| NSSI subsample *without* prior help-seeking | | | | | | | |
| --- | --- | --- | --- | --- | --- | --- | --- |
|  | total  *N*=165 | CG  *n*=68 | INT1  *n*=47 | INT2  *n*=50 | *F* (2,160) | *p* | Pairwise comparisons |
| Potential help-seeking (GHSQ)^a^ |  | | | | | | |
| Professional max. *M (SD)* | 4.49 (1.71) | 4.40 (1.91) | 4.60 (1.36) | 4.52 (1.74) | .23 | .799 |  |
| Informal max. *M (SD)* | 5.84 (1.40) | 5.75 (1.56) | 5.64 (1.28) | 6.14 (1.26) | 1.84 | .163 |  |
| None *M (SD)* | 3.09 (2.03) | 3.00 (2.00) | 3.64 (2.02) | 2.70 (2.01) | 2.93 | .056 | INT1 > INT2 |
| Stigma (USS)^b^ |  | | | | | | |
| Blame *M (SD)* | 4.48 (.61) | 4.44 (.67) | 4.39 (.63) | 4.62 (.47) | 1.98 | .142 |  |
| Distrust *M (SD)* | 4.02 (.68) | 3.90 (.69) | 3.95 (.60) | 4.26 (.70) | 4.50 | .013 | INT2 > CG, INT1 |
| Help-seeking attitudes (IASMHS)^b^ |  | | | | | | |
| Psychological Openness *M (SD)* | 20.68 (4.81) | 20.41 (4.90) | 20.19 (5.05) | 21.50 (4.45) | 1.29 | .277 |  |
| Help-seeking propensity *M (SD)* | 19.76 (5.04) | 20.10 (5.60) | 18.60 (3.83) | 20.38 (5.13) | 2.00 | .139 |  |
| Indifference to stigma *M (SD)* | 23.53 (5.44) | 23.91 (5.01) | 22.00 (6.44) | 24.44 (4.76) | 3.23 | .042 | CG, INT2 > INT1 |
| Video acceptability and transportation^a^ | | | | | | | |
| General likability *M (SD)* | 4.10 (.79) | 3.85 (.83) | 4.30 (.66) | 4.24 (.77) | 5.95 | .003 | INT1, INT2 > CG |
| Comprehensiveness *M (SD)* | 4.81 (.45) | 4.76 (.52) | 4.79 (.46) | 4.90 (.30) | 1.52 | .223 |  |
| Interestingness *M (SD)* | 3.99 (.91) | 3.84 (.99) | 3.94 (.82) | 4.26 (.83) | 3.18 | .044 | INT2 > CG |
| Transportation (TS-SF) *M (SD)* | 4.56 (1.10) | 4.41 (1.12) | 4.76 (.97) | 4.58 (1.17) | 1.64 | .198 |  |

*Note.* *CG* = control group; *GHSQ* = General Help Seeking Questionnaire; *IASMHS* = Inventory of Attitudes toward Seeking Mental Health Services; *INT1* = intervention 1; *INT2* = intervention 2; *TS-SF* = Transportation Scale - Short Form*; USS* = Universal Stigma Scale. ^a^ Higher scores represent a greater level of agreement. ^b^ Higher scores represent more positive attitudes towards mental health issues and help-seeking. Results controlled for MH issue (random factor), age, and number of NSSI events during the past 12 months (SITBI-G) (covariates).

## **Table S9. ANCOVA results and pairwise comparisons for outcomes (problematic alcohol use** sample, participants with prior help-seeking experience only).

| Problematic alcohol use subsample *with* prior help-seeking | | | | | | | |
| --- | --- | --- | --- | --- | --- | --- | --- |
|  | total  *N*=130 | CG  *n*=49 | INT1  *n*=41 | INT2  *n*=40 | *F* (2,125) | *p* | Pairwise comparisons |
| Potential help-seeking (GHSQ)^a^ |  | | | | | | |
| Professional max. *M (SD)* | 5.64 (1.53) | 5.65 (1.60) | 5.85 (1.17) | 5.40 (1.75) | .71 | .495 |  |
| Informal max. *M (SD)* | 6.02 (1.14) | 6.24 (.95) | 5.66 (1.37) | 6.13 (1.02) | 3.03 | .052 | CG > INT1 |
| None *M (SD)* | 2.48 (1.69) | 2.43 (1.78) | 2.59 (1.61) | 2.43 (1.68) | .21 | .809 |  |
| Stigma (USS)^b^ |  | | | | | | |
| Blame *M (SD)* | 4.24 (.64) | 4.26 (.65) | 4.22 (.67) | 4.23 (.62) | .15 | .864 |  |
| Distrust *M (SD)* | 3.27 (.70) | 3.17 (.72) | 3.27 (.65) | 3.40 (.74) | 1.50 | .228 |  |
| Help-seeking attitudes (IASMHS)^b^ |  | | | | | | |
| Psychological Openness *M (SD)* | 22.05 (4.22) | 22.59 (4.21) | 22.34 (3.98) | 21.08 (4.39) | 1.70 | .186 |  |
| Help-seeking propensity *M (SD)* | 23.23 (4.59) | 23.47 (4.09) | 24.07 (4.90) | 22.08 (4.71) | 1.34 | .266 |  |
| Indifference to stigma *M (SD)* | 24.62 (5.31) | 25.02 (5.69) | 24.54 (4.74) | 24.23 (5.49) | .23 | .794 |  |
| Video acceptability and transportation^a^ | | | | | | | |
| General likability *M (SD)* | 3.80 (.76) | 3.76 (.72) | 3.83 (.83) | 3.83 (.75) | .13 | .880 |  |
| Comprehensiveness *M (SD)* | 4.76 (.54) | 4.67 (.63) | 4.85 (.42) | 4.78 (.53) | 1.51 | .226 |  |
| Interestingness *M (SD)* | 3.58 (1.00) | 3.59 (1.02) | 3.56 (1.05) | 3.58 (.93) | .02 | .983 |  |
| Transportation (TS-SF) *M (SD)* | 3.94 (1.18) | 3.96 (1.23) | 3.90 (1.11) | 3.95 (1.22) | .06 | .938 |  |

*Note.* *CG* = control group; *GHSQ* = General Help Seeking Questionnaire; *IASMHS* = Inventory of Attitudes toward Seeking Mental Health Services; *INT1* = intervention 1; *INT2* = intervention 2; *TS-SF* = Transportation Scale - Short Form*; USS* = Universal Stigma Scale. ^a^ Higher scores represent a greater level of agreement. ^b^ Higher scores represent more positive attitudes towards mental health issues and help-seeking. Results controlled for MH issue (random factor), age, and AUDIT-C score (covariates).

## **Table S10. ANCOVA results and pairwise comparisons for outcomes (problematic alcohol use** sample, participants without prior help-seeking experience only).

| Problematic alcohol use subsample *without* prior help-seeking | | | | | | | |
| --- | --- | --- | --- | --- | --- | --- | --- |
|  | total  *N*=149 | CG  *n*=63 | INT1  *n*=43 | INT2  *n*=43 | *F* (2,144) | *p* | Pairwise comparisons |
| Potential help-seeking (GHSQ)^a^ |  | | | | | | |
| Professional max. *M (SD)* | 4.68 (1.74) | 4.59 (1.83) | 4.60 (1.71) | 4.91 (1.65) | .52 | .597 |  |
| Informal max. *M (SD)* | 6.07 (1.28) | 6.13 (1.11) | 5.84 (1.56) | 6.21 (1.19) | .98 | .379 |  |
| None *M (SD)* | 2.70 (1.80) | 2.37 (1.61) | 3.26 (1.95) | 2.65 (1.80) | 2.97 | .055 | INT1 > CG |
| Stigma (USS)^b^ |  | | | | | | |
| Blame *M (SD)* | 3.95 (.76) | 3.81 (.77) | 4.01 (.89) | 4.10 (.56) | 2.47 | .089 |  |
| Distrust *M (SD)* | 3.16 (.77) | 2.99 (.79) | 3.17 (.76) | 3.38 (.72) | 4.00 | .020 | INT2 > CG |
| Help-seeking attitudes (IASMHS)^b^ |  | | | | | | |
| Psychological Openness *M (SD)* | 20.79 (5.02) | 20.46 (5.04) | 20.84 (5.18) | 21.21 (4.89) | .34 | .716 |  |
| Help-seeking propensity *M (SD)* | 20.58 (4.68) | 20.71 (4.78) | 20.02 (4.80) | 20.95 (4.47) | .44 | .646 |  |
| Indifference to stigma *M (SD)* | 23.66 (5.94) | 24.75 (5.09) | 22.02 (6.57) | 23.70 (6.20) | 2.73 | .069 |  |
| Video acceptability and transportation^a^ | | | | | | | |
| General likability *M (SD)* | 3.77 (.87) | 3.81 (.84) | 3.81 (.93) | 3.65 (.87) | .43 | .650 |  |
| Comprehensiveness *M (SD)* | 4.74 (.59) | 4.70 (.64) | 4.70 (.67) | 4.84 (.37) | .90 | .411 |  |
| Interestingness *M (SD)* | 3.62 (.93) | 3.67 (1.03) | 3.65 (.78) | 3.51 (.94) | .34 | .710 |  |
| Transportation (TS-SF) *M (SD)* | 3.85 (1.11) | 3.98 (1.02) | 4.01 (1.31) | 3.50 (.97) | 3.01 | .052 | CG, INT1 > INT2 |

*Note.* *CG* = control group; *GHSQ* = General Help Seeking Questionnaire; *IASMHS* = Inventory of Attitudes toward Seeking Mental Health Services; *INT1* = intervention 1; *INT2* = intervention 2; *TS-SF* = Transportation Scale - Short Form*; USS* = Universal Stigma Scale. ^a^ Higher scores represent a greater level of agreement. ^b^ Higher scores represent more positive attitudes towards mental health issues and help-seeking. Results controlled for MH issue (random factor), age, and AUDIT-C score (covariates).
