# Supplementary material for: The Impact of Video-Based Microinterventions on Attitudes Toward Mental Health and Help Seeking in Youth: Web-Based Randomized Controlled Trial
Source: J Med Internet Res. 2024 Apr 24;26:e54478. doi: 10.2196/54478 (PMC11079770; doi:10.2196/54478)
Supplement: Multimedia Appendix 4 [file jmir_v26i1e54478_app4.pdf]

CONSORT-EHEALTH (V 1.6.1) - Submission/Publication Form

The CONSORT-EHEALTH checklist is intended for authors of randomized trials evaluating web-based and Internet-based applications/interventions, including mobile interventions, electronic games (incl multiplayer games), social media, certain telehealth applications, and other interactive and/or networked electronic applications. Some of the items (e.g. all subitems under item 5 - description of the intervention) may also be applicable for other study designs.

The goal of the CONSORT EHEALTH checklist and guideline is to be
a) a guide for reporting for authors of RCTs,
b) to form a basis for appraisal of an ehealth trial (in terms of validity)

CONSORT-EHEALTH items/subitems are MANDATORY reporting items for studies published in the Journal of Medical Internet Research and other journals / scientific societies endorsing the checklist.

Items numbered 1., 2., 3., 4a., 4b etc are original CONSORT or CONSORT-NPT (non-pharmacologic treatment) items.
Items with Roman numerals (i., ii, iii, iv etc.) are CONSORT-EHEALTH extensions/clarifications.

As the CONSORT-EHEALTH checklist is still considered in a formative stage, we would ask that you also RATE ON A SCALE OF 1-5 how important/useful you feel each item is FOR THE PURPOSE OF THE CHECKLIST and reporting guideline (optional).

Mandatory reporting items are marked with a red *.
In the textboxes, either copy & paste the relevant sections from your manuscript into this form - please include any quotes from your manuscript in QUOTATION MARKS,
or answer directly by providing additional information not in the manuscript, or elaborating on why the item was not relevant for this study.

YOUR ANSWERS WILL BE PUBLISHED AS A SUPPLEMENTARY FILE TO YOUR PUBLICATION IN JMIR AND ARE CONSIDERED PART OF YOUR PUBLICATION (IF ACCEPTED).
Please fill in these questions diligently. Information will not be copyedited, so please use proper spelling and grammar, use correct capitalization, and avoid abbreviations.

DO NOT FORGET TO SAVE AS PDF _AND_ CLICK THE SUBMIT BUTTON SO YOUR ANSWERS ARE IN OUR DATABASE !!!

Citation Suggestion (if you append the pdf as Appendix we suggest to cite this paper in the caption):
Eysenbach G, CONSORT-EHEALTH Group
CONSORT-EHEALTH: Improving and Standardizing Evaluation Reports of Web-based and Mobile Health Interventions
J Med Internet Res 2011;13(4):e126

URL: http://www.jmir.org/2011/4/e126/
doi: 10.2196/jmir.1923
PMID: 22209829

dilemmaqua@gmail.com Konto wechseln

Nicht freigegeben

Entwurf gespeichert

* Gibt eine erforderliche Frage an

Your name *

First Last

Diana Lemmer

Primary Affiliation (short), City, Country *

University of Toronto, Toronto, Canada

Center for Psychotherapy Research, University

Your e-mail address *

abc@gmail.com

lemmer@psyres.de

Title of your manuscript *

Provide the (draft) title of your manuscript.

The Impact of Video-Based Micro-Interventions on Attitudes towards Mental Health and Help-Seeking in Youths: A Web-Based Randomized Controlled Trial.

Name of your App/Software/Intervention *

If there is a short and a long/alternate name, write the short name first and add the long name in brackets.

Not applicable

Evaluated Version (if any)

e.g. "V1", "Release 2017-03-01", "Version 2.0.27913"

Meine Antwort

Language(s) *

What language is the intervention/app in? If multiple languages are available, separate by comma (e.g. "English, French")

German

URL of your Intervention Website or App

e.g. a direct link to the mobile app on app in appstore (itunes, Google Play), or URL of the website. If the intervention is a DVD or hardware, you can also link to an Amazon page.

Meine Antwort

URL of an image/screenshot (optional)

Meine Antwort

Accessibility *

Can an enduser access the intervention presently?

- ◯ access is free and open
- ◯ access only for special usergroups, not open
- ◯ access is open to everyone, but requires payment/subscription/in-app purchases
- ◉ app/intervention no longer accessible
- ◯ Sonstiges:

Primary Medical Indication/Disease/Condition *

e.g. "Stress", "Diabetes", or define the target group in brackets after the condition, e.g. "Autism (Parents of children with)", "Alzheimers (Informal Caregivers of)"

None required. A general youth population was

Primary Outcomes measured in trial *

comma-separated list of primary outcomes reported in the trial

potential professional help-seeking (General H

Secondary/other outcomes

Are there any other outcomes the intervention is expected to affect?

potential help-seeking (none, informal), stigma, attitudes towards mental health and help-seeking, acceptability, transportation/ immersiveness

Recommended "Dose" *

What do the instructions for users say on how often the app should be used?

- ○ Approximately Daily
- ○ Approximately Weekly
- ○ Approximately Monthly
- ○ Approximately Yearly
- ○ "as needed"
- ◉ Sonstiges: Not applicable

Approx. Percentage of Users (starters) still using the app as recommended after 3 months *

- ○ unknown / not evaluated
- ○ 0-10%
- ○ 11-20%
- ○ 21-30%
- ○ 31-40%
- ○ 41-50%
- ○ 51-60%
- ○ 61-70%
- ○ 71%-80%
- ○ 81-90%
- ○ 91-100%
- ◉ Sonstiges: Intervention videos are not publicly accessible anymore.

Overall, was the app/intervention effective? *

- ( ) yes: all primary outcomes were significantly better in intervention group vs control
- ( ) partly: SOME primary outcomes were significantly better in intervention group vs control
- ( ) no statistically significant difference between control and intervention
- ( ) potentially harmful: control was significantly better than intervention in one or more outcomes
- (x) inconclusive: more research is needed
- ( ) Sonstiges:

Article Preparation Status/Stage *

At which stage in your article preparation are you currently (at the time you fill in this form)

- ( ) not submitted yet - in early draft status
- ( ) not submitted yet - in late draft status, just before submission
- ( ) submitted to a journal but not reviewed yet
- (x) submitted to a journal and after receiving initial reviewer comments
- ( ) submitted to a journal and accepted, but not published yet
- ( ) published
- ( ) Sonstiges:

Journal *

If you already know where you will submit this paper (or if it is already submitted), please provide the journal name (if it is not JMIR, provide the journal name under "other")

- ○ not submitted yet / unclear where I will submit this
- ◉ Journal of Medical Internet Research (JMIR)
- ○ JMIR mHealth and UHealth
- ○ JMIR Serious Games
- ○ JMIR Mental Health
- ○ JMIR Public Health
- ○ JMIR Formative Research
- ○ Other JMIR sister journal
- ○ Sonstiges:

Is this a full powered effectiveness trial or a pilot/feasibility trial? *

- ○ Pilot/feasibility
- ◉ Fully powered

Manuscript tracking number *

If this is a JMIR submission, please provide the manuscript tracking number under "other" (The ms tracking number can be found in the submission acknowledgement email, or when you login as author in JMIR. If the paper is already published in JMIR, then the ms tracking number is the four-digit number at the end of the DOI, to be found at the bottom of each published article in JMIR)

- ○ no ms number (yet) / not (yet) submitted to / published in JMIR
- ◉ Sonstiges: ms #54478

TITLE AND ABSTRACT

1a) TITLE: Identification as a randomized trial in the title

1a) Does your paper address CONSORT item 1a? *

I.e does the title contain the phrase "Randomized Controlled Trial"? (if not, explain the reason under "other")

- (selected) yes
- ( ) Sonstiges:

1a-i) Identify the mode of delivery in the title

Identify the mode of delivery. Preferably use “web-based” and/or “mobile” and/or “electronic game” in the title. Avoid ambiguous terms like “online”, “virtual”, “interactive”. Use “Internet-based” only if Intervention includes non-web-based Internet components (e.g. email), use “computer-based” or “electronic” only if offline products are used. Use “virtual” only in the context of “virtual reality” (3-D worlds). Use “online” only in the context of “online support groups”. Complement or substitute product names with broader terms for the class of products (such as “mobile” or “smart phone” instead of “iphone”), especially if the application runs on different platforms.

	1	2	3	4	5	
subitem not at all important	○	○	○	●	○	essential

Auswahl löschen

Does your paper address subitem 1a-i? *

Copy and paste relevant sections from manuscript title (include quotes in quotation marks "like this" to indicate direct quotes from your manuscript), or elaborate on this item by providing additional information not in the ms, or briefly explain why the item is not applicable/relevant for your study

"The Impact of Video-Based Micro-Interventions on Attitudes towards Mental Health and Help-Seeking in Youths: A Web-Based Randomized Controlled Trial."

1a-ii) Non-web-based components or important co-interventions in title

Mention non-web-based components or important co-interventions in title, if any (e.g., “with telephone support”).

	1	2	3	4	5	
subitem not at all important	○	○	◉	○	○	essential

Auswahl löschen

Does your paper address subitem 1a-ii?

Copy and paste relevant sections from manuscript title (include quotes in quotation marks "like this" to indicate direct quotes from your manuscript), or elaborate on this item by providing additional information not in the ms, or briefly explain why the item is not applicable/relevant for your study

Meine Antwort

1a-iii) Primary condition or target group in the title

Mention primary condition or target group in the title, if any (e.g., “for children with Type I Diabetes”) Example: A Web-based and Mobile Intervention with Telephone Support for Children with Type I Diabetes: Randomized Controlled Trial

	1	2	3	4	5	
subitem not at all important	○	○	○	○	◉	essential

Auswahl löschen

Does your paper address subitem 1a-iii? *

Copy and paste relevant sections from manuscript title (include quotes in quotation marks "like this" to indicate direct quotes from your manuscript), or elaborate on this item by providing additional information not in the ms, or briefly explain why the item is not applicable/relevant for your study

"The Impact of Video-Based Micro-Interventions on Attitudes towards Mental Health and Help-Seeking in Youths: A Web-Based Randomized Controlled Trial."

1b) ABSTRACT: Structured summary of trial design, methods, results, and conclusions

NPT extension: Description of experimental treatment, comparator, care providers, centers, and blinding status.

1b-i) Key features/functionalities/components of the intervention and comparator in the METHODS section of the ABSTRACT

Mention key features/functionalities/components of the intervention and comparator in the abstract. If possible, also mention theories and principles used for designing the site. Keep in mind the needs of systematic reviewers and indexers by including important synonyms. (Note: Only report in the abstract what the main paper is reporting. If this information is missing from the main body of text, consider adding it)

	1	2	3	4	5	
subitem not at all important	○	○	○	◉	○	essential

Auswahl löschen

Does your paper address subitem 1b-i? *

Copy and paste relevant sections from the manuscript abstract (include quotes in quotation marks "like this" to indicate direct quotes from your manuscript), or elaborate on this item by providing additional information not in the ms, or briefly explain why the item is not applicable/relevant for your study

"This study was entirely web-based and open-access. The interventions addressed five MH problems: generalized anxiety disorder (GAD), depression, bulimia, non-suicidal self-injury (NSSI), and problematic alcohol use. Intervention 1 (INT1) aimed at destigmatization and improving MH literacy, whereas intervention 2 (INT2) aimed to induce positive outcome expectancies towards professional help-seeking." (...) "After the presentation of a video vignette, no further video was shown to the control group, while a second, short intervention video was presented to the INT1 and the INT2 groups."

1b-ii) Level of human involvement in the METHODS section of the ABSTRACT

Clarify the level of human involvement in the abstract, e.g., use phrases like “fully automated” vs. “therapist/nurse/care provider/physician-assisted” (mention number and expertise of providers involved, if any). (Note: Only report in the abstract what the main paper is reporting. If this information is missing from the main body of text, consider adding it)

	1	2	3	4	5	
subitem not at all important	○	○	◉	○	○	essential

Auswahl löschen

Does your paper address subitem 1b-ii?

Copy and paste relevant sections from the manuscript abstract (include quotes in quotation marks "like this" to indicate direct quotes from your manuscript), or elaborate on this item by providing additional information not in the ms, or briefly explain why the item is not applicable/relevant for your study

"(...) a final sample of N=1394 participants (57%) aged 14 to 29 years with complete data and sufficient durations of stay on the video pages were randomized in a fully automated manner to one of the five MH problems and one of three conditions (control, INT1, INT2) in a permuted block design."

1b-iii) Open vs. closed, web-based (self-assessment) vs. face-to-face assessments in the METHODS section of the ABSTRACT

Mention how participants were recruited (online vs. offline), e.g., from an open access website or from a clinic or a closed online user group (closed usergroup trial), and clarify if this was a purely web-based trial, or there were face-to-face components (as part of the intervention or for assessment). Clearly say if outcomes were self-assessed through questionnaires (as common in web-based trials). Note: In traditional offline trials, an open trial (open-label trial) is a type of clinical trial in which both the researchers and participants know which treatment is being administered. To avoid confusion, use “blinded” or “unblinded” to indicated the level of blinding instead of “open”, as “open” in web-based trials usually refers to “open access” (i.e. participants can self-enrol). (Note: Only report in the abstract what the main paper is reporting. If this information is missing from the main body of text, consider adding it)

	1	2	3	4	5	
subitem not at all important	○	○	◉	○	○	essential

Auswahl löschen

Does your paper address subitem 1b-iii?

Copy and paste relevant sections from the manuscript abstract (include quotes in quotation marks "like this" to indicate direct quotes from your manuscript), or elaborate on this item by providing additional information not in the ms, or briefly explain why the item is not applicable/relevant for your study

"This study was entirely web-based and open-access."
"Intervention effects on self-reported potential professional help-seeking (primary outcome), stigma, and attitudes towards help-seeking were examined with analyses of covariance across and within the five MH problems."

1b-iv) RESULTS section in abstract must contain use data

Report number of participants enrolled/assessed in each group, the use/uptake of the intervention (e.g., attrition/adherence metrics, use over time, number of logins etc.), in addition to primary/secondary outcomes. (Note: Only report in the abstract what the main paper is reporting. If this information is missing from the main body of text, consider adding it)

	1	2	3	4	5	
subitem not at all important	○	○	◉	○	○	essential

Auswahl löschen

Does your paper address subitem 1b-iv?

Copy and paste relevant sections from the manuscript abstract (include quotes in quotation marks "like this" to indicate direct quotes from your manuscript), or elaborate on this item by providing additional information not in the ms, or briefly explain why the item is not applicable/relevant for your study

"Out of N=2435 participants who commenced participation, a final sample of N=1394 participants (57%) aged 14 to 29 years with complete data and sufficient durations of stay on the video pages were randomized in a fully automated manner to one of the five MH problems and one of three conditions (control, INT1, INT2) in a permuted block design."

1b-v) CONCLUSIONS/DISCUSSION in abstract for negative trials

Conclusions/Discussions in abstract for negative trials: Discuss the primary outcome - if the trial is negative (primary outcome not changed), and the intervention was not used, discuss whether negative results are attributable to lack of uptake and discuss reasons. (Note: Only report in the abstract what the main paper is reporting. If this information is missing from the main body of text, consider adding it)

	1	2	3	4	5	
subitem not at all important	○	○	◉	○	○	essential

Auswahl löschen

Does your paper address subitem 1b-v?

Copy and paste relevant sections from the manuscript abstract (include quotes in quotation marks "like this" to indicate direct quotes from your manuscript), or elaborate on this item by providing additional information not in the ms, or briefly explain why the item is not applicable/relevant for your study

Meine Antwort

INTRODUCTION

2a) In INTRODUCTION: Scientific background and explanation of rationale

2a-i) Problem and the type of system/solution

Describe the problem and the type of system/solution that is object of the study: intended as stand-alone intervention vs. incorporated in broader health care program? Intended for a particular patient population? Goals of the intervention, e.g., being more cost-effective to other interventions, replace or complement other solutions? (Note: Details about the intervention are provided in “Methods” under 5)

	1	2	3	4	5	
subitem not at all important	○	◉	○	○	○	essential

Auswahl löschen

Does your paper address subitem 2a-i? *

Copy and paste relevant sections from the manuscript (include quotes in quotation marks "like this" to indicate direct quotes from your manuscript), or elaborate on this item by providing additional information not in the ms, or briefly explain why the item is not applicable/relevant for your study

"Clearly, there is a need for more research in this area, particularly with respect to digital brief and micro-interventions (i.e. highly focused in-the-moment interventions with a narrower scope and time-frame than standard interventions [38]) which allow for a flexible, easily-accessible, scalable, and efficient delivery of MH content."

"Concerning the theoretical foundation of interventions, few studies have investigated help-seeking promoting strategies which were explicitly based on the premises of health behavior models."

"Building on the findings of previous research, this study investigates the short-term effectiveness of two brief animated video interventions to promote potential professional help-seeking in a general sample of adolescents and young adults aged 14 to 29 years with a web-based RCT approach. Both interventions aimed to improve participants' willingness to seek professional help (i.e. psychotherapists, psychiatrists, and counselling services) for five MH problems (generalized anxiety disorder (GAD), depression, bulimia, non-suicidal self-injury (NSSI), problematic alcohol use). The inclusion of various MH problems allowed for the investigation of potential differential effects."

"Within the framework of this study, the videos were evaluated as stand-alone interventions. They were not developed to replace existing interventions. However, in case of favorable outcomes, they have the potential to complement existing healthcare services."

2a-ii) Scientific background, rationale: What is known about the (type of) system

Scientific background, rationale: What is known about the (type of) system that is the object of the study (be sure to discuss the use of similar systems for other conditions/diagnoses, if appropiate), motivation for the study, i.e. what are the reasons for and what is the context for this specific study, from which stakeholder viewpoint is the study performed, potential impact of findings [2]. Briefly justify the choice of the comparator.

	1	2	3	4	5	
subitem not at all important	○	○	◉	○	○	essential

Auswahl löschen

Does your paper address subitem 2a-ii? *

Copy and paste relevant sections from the manuscript (include quotes in quotation marks "like this" to indicate direct quotes from your manuscript), or elaborate on this item by providing additional information not in the ms, or briefly explain why the item is not applicable/relevant for your study

Scientific background is elaborated throughout the introduction section.
Justification of choice of the comparator: "The interventions were both compared to each other and a non-intervention control group, where participants were presented with a stand-alone video vignette without an additional intervention video. This approach was both chosen due to contextual (i.e. vignette characters were described to experience difficulties in several life domains, thus additional control videos referring to the vignettes were unfeasible) and practical considerations (i.e. no creation of ten additional videos necessary)."

2b) In INTRODUCTION: Specific objectives or hypotheses

Does your paper address CONSORT subitem 2b? *

Copy and paste relevant sections from the manuscript (include quotes in quotation marks "like this" to indicate direct quotes from your manuscript), or elaborate on this item by providing additional information not in the ms, or briefly explain why the item is not applicable/relevant for your study

"This study had the following objectives:
1. To investigate the short-term effectiveness of the two interventions in the promotion of potential MH help-seeking (professional and informal), whereby self-reported professional help-seeking was defined as primary outcome.
2. To investigate the interventions’ effectiveness in the improvement of self-reported attitudes towards MH problems and MH service use (stigmatization, attitudes towards seeking MH services).
3. To evaluate the interventions’ self-reported acceptability."

METHODS

3a) Description of trial design (such as parallel, factorial) including allocation ratio

Does your paper address CONSORT subitem 3a? *

Copy and paste relevant sections from the manuscript (include quotes in quotation marks "like this" to indicate direct quotes from your manuscript), or elaborate on this item by providing additional information not in the ms, or briefly explain why the item is not applicable/relevant for your study

"This anonymous, fully automated web-based parallel group exploratory RCT compared the effects of INT1 (psychoeducational intervention) and INT2 (positive consequences of help-seeking) against the control group (CG; no further video after case vignette) with regard to potential help-seeking, attitudes towards help-seeking, and stigma. The design comprised 15 conditions in total (5 MH problems x 3 interventional conditions). Randomization was stratified by gender and implemented with a permuted block design (block sizes: 15, 30)."

3b) Important changes to methods after trial commencement (such as eligibility criteria), with reasons

Does your paper address CONSORT subitem 3b? *

Copy and paste relevant sections from the manuscript (include quotes in quotation marks "like this" to indicate direct quotes from your manuscript), or elaborate on this item by providing additional information not in the ms, or briefly explain why the item is not applicable/relevant for your study

"Deviations from the protocol
In the beginning of recruitment, the upper age limit was raised from originally 25 to 29 years due to the above-mentioned findings of prior research."

3b-i) Bug fixes, Downtimes, Content Changes

Bug fixes, Downtimes, Content Changes: ehealth systems are often dynamic systems. A description of changes to methods therefore also includes important changes made on the intervention or comparator during the trial (e.g., major bug fixes or changes in the functionality or content) (5-iii) and other “unexpected events” that may have influenced study design such as staff changes, system failures/downtimes, etc. [2].

	1	2	3	4	5	
subitem not at all important	○	○	◉	○	○	essential

Auswahl löschen

Does your paper address subitem 3b-i?

Copy and paste relevant sections from the manuscript (include quotes in quotation marks "like this" to indicate direct quotes from your manuscript), or elaborate on this item by providing additional information not in the ms, or briefly explain why the item is not applicable/relevant for your study

Meine Antwort

4a) Eligibility criteria for participants

Does your paper address CONSORT subitem 4a? *

Copy and paste relevant sections from the manuscript (include quotes in quotation marks "like this" to indicate direct quotes from your manuscript), or elaborate on this item by providing additional information not in the ms, or briefly explain why the item is not applicable/relevant for your study

"Youths aged between 14 and 29 years with sufficient German language skills were eligible for participation. The age of 14 years is widely accepted as appropriate to provide informed consent for medical decisions and the participation in studies [54, 55]. The upper age limit of 29 years aligns with the definition of emerging adulthood, a separate life stage between adolescence and adulthood [56, 57]."

4a-i) Computer / Internet literacy

Computer / Internet literacy is often an implicit “de facto” eligibility criterion - this should be explicitly clarified.

	1	2	3	4	5	
subitem not at all important	○	◉	○	○	○	essential

Auswahl löschen

Does your paper address subitem 4a-i?

Copy and paste relevant sections from the manuscript (include quotes in quotation marks "like this" to indicate direct quotes from your manuscript), or elaborate on this item by providing additional information not in the ms, or briefly explain why the item is not applicable/relevant for your study

Meine Antwort

4a-ii) Open vs. closed, web-based vs. face-to-face assessments:

Open vs. closed, web-based vs. face-to-face assessments: Mention how participants were recruited (online vs. offline), e.g., from an open access website or from a clinic, and clarify if this was a purely web-based trial, or there were face-to-face components (as part of the intervention or for assessment), i.e., to what degree got the study team to know the participant. In online-only trials, clarify if participants were quasi-anonymous and whether having multiple identities was possible or whether technical or logistical measures (e.g., cookies, email confirmation, phone calls) were used to detect/prevent these.

	1	2	3	4	5	
subitem not at all important	○	○	○	◉	○	essential

Auswahl löschen

Does your paper address subitem 4a-ii? *

Copy and paste relevant sections from the manuscript (include quotes in quotation marks "like this" to indicate direct quotes from your manuscript), or elaborate on this item by providing additional information not in the ms, or briefly explain why the item is not applicable/relevant for your study

"This anonymous, fully automated web-based parallel group exploratory RCT compared the effects of INT1 (psychoeducational intervention) and INT2 (positive consequences of help-seeking) against the control group (CG; no further video after case vignette) with regard to potential help-seeking, attitudes towards help-seeking, and stigma."
"This study was conducted in an open access, voluntary web-based setting. A website has been established to provide study information and enable participation."

4a-iii) Information giving during recruitment

Information given during recruitment. Specify how participants were briefed for recruitment and in the informed consent procedures (e.g., publish the informed consent documentation as appendix, see also item X26), as this information may have an effect on user self-selection, user expectation and may also bias results.

	1	2	3	4	5	
subitem not at all important	○	○	◉	○	○	essential

Auswahl löschen

Does your paper address subitem 4a-iii?

Copy and paste relevant sections from the manuscript (include quotes in quotation marks "like this" to indicate direct quotes from your manuscript), or elaborate on this item by providing additional information not in the ms, or briefly explain why the item is not applicable/relevant for your study

"Prior to their participation, youths received detailed information about the aims, scope, procedures, data processing, and data storage of the study on the website. Participants were informed that they would be randomly assigned to one out of five health problems and one out of three video versions. They were not informed about the specific health issues or the conditions' details prior to participation. (...) After study completion, participants were debriefed in writing about the objectives on the study website. The debriefing form also included contact information of formal help services."

4b) Settings and locations where the data were collected

Does your paper address CONSORT subitem 4b? *

Copy and paste relevant sections from the manuscript (include quotes in quotation marks "like this" to indicate direct quotes from your manuscript), or elaborate on this item by providing additional information not in the ms, or briefly explain why the item is not applicable/relevant for your study

"Data were collected at the Center for Psychotherapy Research, Heidelberg."

4b-i) Report if outcomes were (self-)assessed through online questionnaires

Clearly report if outcomes were (self-)assessed through online questionnaires (as common in web-based trials) or otherwise.

	1	2	3	4	5	
subitem not at all important	○	○	○	◉	○	essential

Auswahl löschen

Does your paper address subitem 4b-i? *

Copy and paste relevant sections from the manuscript (include quotes in quotation marks "like this" to indicate direct quotes from your manuscript), or elaborate on this item by providing additional information not in the ms, or briefly explain why the item is not applicable/relevant for your study

"All measures were self-reported."

4b-ii) Report how institutional affiliations are displayed

Report how institutional affiliations are displayed to potential participants [on ehealth media], as affiliations with prestigious hospitals or universities may affect volunteer rates, use, and reactions with regards to an intervention.(Not a required item – describe only if this may bias results)

	1	2	3	4	5	
subitem not at all important	◉	○	○	○	○	essential

Auswahl löschen

Does your paper address subitem 4b-ii?

Copy and paste relevant sections from the manuscript (include quotes in quotation marks "like this" to indicate direct quotes from your manuscript), or elaborate on this item by providing additional information not in the ms, or briefly explain why the item is not applicable/relevant for your study

Meine Antwort

5) The interventions for each group with sufficient details to allow replication, including how and when they were actually administered

5-i) Mention names, credential, affiliations of the developers, sponsors, and owners

Mention names, credential, affiliations of the developers, sponsors, and owners [6] (if authors/evaluators are owners or developer of the software, this needs to be declared in a “Conflict of interest” section or mentioned elsewhere in the manuscript).

	1	2	3	4	5	
subitem not at all important	○	○	◉	○	○	essential

Auswahl löschen

Does your paper address subitem 5-i?

Copy and paste relevant sections from the manuscript (include quotes in quotation marks "like this" to indicate direct quotes from your manuscript), or elaborate on this item by providing additional information not in the ms, or briefly explain why the item is not applicable/relevant for your study

Meine Antwort

5-ii) Describe the history/development process

Describe the history/development process of the application and previous formative evaluations (e.g., focus groups, usability testing), as these will have an impact on adoption/use rates and help with interpreting results.

	1	2	3	4	5	
subitem not at all important	○	○	◉	○	○	essential

Auswahl löschen

Does your paper address subitem 5-ii?

Copy and paste relevant sections from the manuscript (include quotes in quotation marks "like this" to indicate direct quotes from your manuscript), or elaborate on this item by providing additional information not in the ms, or briefly explain why the item is not applicable/relevant for your study

See Methods -> Procedure -> Experimental conditions and materials.
"The interventional strategies were applied with short animated videos. The videos were created with the Pro+ version of the online animation tool Powtoon [67]. Each research group involved in this study prepared materials for one of the five MH problems based on their respective field of expertise."
"A subset of the videos was pre-tested between July and September 2020 with a convenience sample of N=9 youths (Mage=18.56, SD=3.74, range: 14-24 years, 1/3 male) who confirmed comprehensibility and overall acceptability."
"The vignettes introduced the characters to the viewers in a third-person perspective and described their challenges in their everyday lives due to their MH conditions (e.g. difficult emotions and cognitions, physical symptoms, social and school-related issues). The accurate diagnostic labels were not presented in the vignettes [68]. Vignette duration ranged from 02:19 to 02:47 minutes (M=02:29, SD=0:11). The bulimia vignettes were developed first. They were inspired by the vignettes of Mond et al. (2010) [69], and adapted in accordance with ICD-10 and DSM-5 diagnostic criteria as well as further literature on the symptomatology and psychological strain of bulimia [70]. The bulimia vignettes then served as a template for the other four MH problems."
"INT1 aimed to improve MH literacy and decrease stigmatization through the presentation of psychoeducational information in order to encourage help-seeking. These intervention videos first presented the correct diagnostic label, prevalence rates, and core symptoms of the condition shown in the vignette. Next, five destigmatizing and psychoeducational facts about the respective condition were presented (e.g. "Bulimia is a serious illness and not a lifestyle.") which were inspired by Bulik [71]. The videos then presented treatment options, information about potential challenges in professional help-seeking, and encouraging statements about the benefits of professional MH support. INT1 video durations ranged from 04:00 to 05:00 minutes (M=04:27, SD=0:21). The information provided in these intervention videos was based on epidemiological, etiological, diagnostic, barrier-related, and interventional findings on the respective MH problems [e.g. for bulimia: 71, 72, 73]."
"The second strategy (INT2) was based on the premises of the Health Action Process Approach (HAPA) [48]. INT2 was designed to induce positive outcome expectancies of professional help-seeking through the continuation of Paul's and Paula's stories. (...) These interventions were designed in accordance with previous literature on the therapeutic process in MH conditions, including treatment expectations, experiences, and consequences [e.g. 74]."

5-iii) Revisions and updating

Revisions and updating. Clearly mention the date and/or version number of the application/intervention (and comparator, if applicable) evaluated, or describe whether the intervention underwent major changes during the evaluation process, or whether the development and/or content was “frozen” during the trial. Describe dynamic components such as news feeds or changing content which may have an impact on the replicability of the intervention (for unexpected events see item 3b).

	1	2	3	4	5	
subitem not at all important	○	○	◉	○	○	essential

Auswahl löschen

Does your paper address subitem 5-iii?

Copy and paste relevant sections from the manuscript (include quotes in quotation marks "like this" to indicate direct quotes from your manuscript), or elaborate on this item by providing additional information not in the ms, or briefly explain why the item is not applicable/relevant for your study

Meine Antwort

5-iv) Quality assurance methods

Provide information on quality assurance methods to ensure accuracy and quality of information provided [1], if applicable.

	1	2	3	4	5	
subitem not at all important	○	◉	○	○	○	essential

Auswahl löschen

Does your paper address subitem 5-iv?

Copy and paste relevant sections from the manuscript (include quotes in quotation marks "like this" to indicate direct quotes from your manuscript), or elaborate on this item by providing additional information not in the ms, or briefly explain why the item is not applicable/relevant for your study

Meine Antwort

5-v) Ensure replicability by publishing the source code, and/or providing screenshots/screen-capture video, and/or providing flowcharts of the algorithms used

Ensure replicability by publishing the source code, and/or providing screenshots/screen-capture video, and/or providing flowcharts of the algorithms used. Replicability (i.e., other researchers should in principle be able to replicate the study) is a hallmark of scientific reporting.

	1	2	3	4	5	
subitem not at all important	○	○	◉	○	○	essential

Auswahl löschen

Does your paper address subitem 5-v?

Copy and paste relevant sections from the manuscript (include quotes in quotation marks "like this" to indicate direct quotes from your manuscript), or elaborate on this item by providing additional information not in the ms, or briefly explain why the item is not applicable/relevant for your study

Meine Antwort

5-vi) Digital preservation

Digital preservation: Provide the URL of the application, but as the intervention is likely to change or disappear over the course of the years; also make sure the intervention is archived (Internet Archive, webcitation.org, and/or publishing the source code or screenshots/videos alongside the article). As pages behind login screens cannot be archived, consider creating demo pages which are accessible without login.

	1	2	3	4	5	
subitem not at all important	○	◉	○	○	○	essential

Auswahl löschen

Does your paper address subitem 5-vi?

Copy and paste relevant sections from the manuscript (include quotes in quotation marks "like this" to indicate direct quotes from your manuscript), or elaborate on this item by providing additional information not in the ms, or briefly explain why the item is not applicable/relevant for your study

Meine Antwort

5-vii) Access

Access: Describe how participants accessed the application, in what setting/context, if they had to pay (or were paid) or not, whether they had to be a member of specific group. If known, describe how participants obtained "access to the platform and Internet" [1]. To ensure access for editors/reviewers/readers, consider to provide a "backdoor" login account or demo mode for reviewers/readers to explore the application (also important for archiving purposes, see vi).

	1	2	3	4	5	
subitem not at all important	○	○	○	◉	○	essential

Auswahl löschen

Does your paper address subitem 5-vii? *

Copy and paste relevant sections from the manuscript (include quotes in quotation marks "like this" to indicate direct quotes from your manuscript), or elaborate on this item by providing additional information not in the ms, or briefly explain why the item is not applicable/relevant for your study

"This study was conducted in an open access, voluntary web-based setting. A website has been established to provide study information and enable participation."

5-viii) Mode of delivery, features/functionalities/components of the intervention and comparator, and the theoretical framework

Describe mode of delivery, features/functionalities/components of the intervention and comparator, and the theoretical framework [6] used to design them (instructional strategy [1], behaviour change techniques, persuasive features, etc., see e.g., [7, 8] for terminology). This includes an in-depth description of the content (including where it is coming from and who developed it) [1]," whether [and how] it is tailored to individual circumstances and allows users to track their progress and receive feedback" [6]. This also includes a description of communication delivery channels and – if computer-mediated communication is a component – whether communication was synchronous or asynchronous [6]. It also includes information on presentation strategies [1], including page design principles, average amount of text on pages, presence of hyperlinks to other resources, etc. [1].

	1	2	3	4	5	
subitem not at all important	○	○	○	○	◉	essential

Auswahl löschen

Does your paper address subitem 5-viii? *

Copy and paste relevant sections from the manuscript (include quotes in quotation marks "like this" to indicate direct quotes from your manuscript), or elaborate on this item by providing additional information not in the ms, or briefly explain why the item is not applicable/relevant for your study

"This study was conducted in an open access, voluntary web-based setting. (...) Study duration amounted to approximately 30 minutes. Participants were first asked to complete sociodemographic and screening questionnaires, were then randomly assigned to one of the 15 experimental conditions, and were finally presented with the outcome questionnaires."

See Methods -> Procedure -> Experimental conditions and materials.
"The interventional strategies were applied with short animated videos. The videos were created with the Pro+ version of the online animation tool Powtoon [67]. Each research group involved in this study prepared materials for one of the five MH problems based on their respective field of expertise. The materials were structured in a similar fashion across MH problems. The main characters Paul and Paula were introduced as 16-year old students in each condition. In total, 30 videos have been created: 5 MH problems x 2 main character genders x 3 video types. Participants in the control conditions only viewed a vignette, whilst participants in both intervention groups each viewed one additional video (either INT1 or INT2)."
"Vignettes. All participants viewed a case vignette. Each vignette depicted the respective main character who is affected by one out of five MH problems (GAD, depression, bulimia, NSSI, problematic alcohol use). The vignettes introduced the characters to the viewers in a third-person perspective and described their challenges in their everyday lives due to their MH conditions (e.g. difficult emotions and cognitions, physical symptoms, social and school-related issues). The accurate diagnostic labels were not presented in the vignettes [68]. Vignette duration ranged from 02:19 to 02:47 minutes (M=02:29, SD=0:11). The bulimia vignettes were developed first. They were inspired by the vignettes of Mond et al. (2010) [69], and adapted in accordance with ICD-10 and DSM-5 diagnostic criteria as well as further literature on the symptomatology and psychological strain of bulimia [70]. The bulimia vignettes then served as a template for the other four MH problems.
Intervention 1. INT1 aimed to improve MH literacy and decrease stigmatization through the presentation of psychoeducational information in order to encourage help-seeking. These intervention videos first presented the correct diagnostic label, prevalence rates, and core symptoms of the condition shown in the vignette. Next, five destigmatizing and psychoeducational facts about the respective condition were presented (e.g. “Bulimia is a serious illness and not a lifestyle.”) which were inspired by Bulik [71]. The videos then presented treatment options, information about potential challenges in professional help-seeking, and encouraging statements about the benefits of professional MH support. INT1 video durations ranged from 04:00 to 05:00 minutes (M=04:27, SD=0:21). The information provided in these intervention videos was based on epidemiological, etiological, diagnostic, barrier-related, and interventional findings on the respective MH problems [e.g. for bulimia: 71, 72, 73].
Intervention 2. The second strategy (INT2) was based on the premises of the Health Action Process Approach (HAPA) [48]. INT2 was designed to induce positive outcome expectancies of professional help-seeking through the continuation of Paul’s and Paula’s stories. The videos showed the main characters one year after their initial situation as described in the vignettes. INT2 videos first demonstrated the help-seeking process of the main characters in a retrospective fashion. Encouraged by their teachers, friends, or parents, the main characters sought and received professional support by a psychotherapist. The psychotherapist’s gender matched the respective gender of the main character. The videos showed how the psychotherapist informed the main character about the correct diagnostic label of their condition and shortly portrayed the therapeutic process. The process included initial difficulties of the main character, such as feelings of insecurity about disclosing their experiences to their therapist, which were resolved over time, and the main characters became invested in their psychotherapy. Then, five positive consequences of psychotherapy were presented, such as decreased impairment and an improved quality of life. The videos

[illegible]
ended with the notion that the main character was still facing occasional difficulties, but substantial improvements in overall well-being and satisfaction with their decision to seek help were emphasized. INT2 video durations ranged from 04:01 to 04:29 minutes (M=04:15, SD=0:14). These interventions were designed in accordance with previous literature on the therapeutic process in MH conditions, including treatment expectations, experiences, and consequences [e.g. 74]."

5-ix) Describe use parameters

Describe use parameters (e.g., intended “doses” and optimal timing for use). Clarify what instructions or recommendations were given to the user, e.g., regarding timing, frequency, heaviness of use, if any, or was the intervention used ad libitum.

	1	2	3	4	5	
subitem not at all important	○	○	◉	○	○	essential

Auswahl löschen

Does your paper address subitem 5-ix?

Copy and paste relevant sections from the manuscript (include quotes in quotation marks "like this" to indicate direct quotes from your manuscript), or elaborate on this item by providing additional information not in the ms, or briefly explain why the item is not applicable/relevant for your study

Meine Antwort

5-x) Clarify the level of human involvement

Clarify the level of human involvement (care providers or health professionals, also technical assistance) in the e-intervention or as co-intervention (detail number and expertise of professionals involved, if any, as well as “type of assistance offered, the timing and frequency of the support, how it is initiated, and the medium by which the assistance is delivered”. It may be necessary to distinguish between the level of human involvement required for the trial, and the level of human involvement required for a routine application outside of a RCT setting (discuss under item 21 – generalizability).

	1	2	3	4	5	
subitem not at all important	○	○	○	●	○	essential

Auswahl löschen

Does your paper address subitem 5-x?

Copy and paste relevant sections from the manuscript (include quotes in quotation marks "like this" to indicate direct quotes from your manuscript), or elaborate on this item by providing additional information not in the ms, or briefly explain why the item is not applicable/relevant for your study

"This anonymous, fully automated web-based parallel group exploratory RCT compared the effects of ..."

5-xi) Report any prompts/reminders used

Report any prompts/reminders used: Clarify if there were prompts (letters, emails, phone calls, SMS) to use the application, what triggered them, frequency etc. It may be necessary to distinguish between the level of prompts/reminders required for the trial, and the level of prompts/reminders for a routine application outside of a RCT setting (discuss under item 21 – generalizability).

	1	2	3	4	5	
subitem not at all important	○	○	●	○	○	essential

Auswahl löschen

Does your paper address subitem 5-xi? *

Copy and paste relevant sections from the manuscript (include quotes in quotation marks "like this" to indicate direct quotes from your manuscript), or elaborate on this item by providing additional information not in the ms, or briefly explain why the item is not applicable/relevant for your study

Not applicable. We assessed video-based micro-interventions in a single session.

5-xii) Describe any co-interventions (incl. training/support)

Describe any co-interventions (incl. training/support): Clearly state any interventions that are provided in addition to the targeted eHealth intervention, as ehealth intervention may not be designed as stand-alone intervention. This includes training sessions and support [1]. It may be necessary to distinguish between the level of training required for the trial, and the level of training for a routine application outside of a RCT setting (discuss under item 21 – generalizability.

	1	2	3	4	5	
subitem not at all important	○	○	○	◉	○	essential

Auswahl löschen

Does your paper address subitem 5-xii? *

Copy and paste relevant sections from the manuscript (include quotes in quotation marks "like this" to indicate direct quotes from your manuscript), or elaborate on this item by providing additional information not in the ms, or briefly explain why the item is not applicable/relevant for your study

(In Introduction) "Within the framework of this study, the videos were evaluated as stand-alone interventions. They were not developed to replace existing interventions. However, in case of favorable outcomes, they have the potential to complement existing healthcare services."

We recruited a general youth sample, of which 19.6% were utilizing professional mental health services at the time of recruitment. These were, however, provided outside of the study context. No other interventions were provided within the context of the study.

"45% were help-seekers, i.e. they utilized professional MH services at the time of or prior to data collection."

6a) Completely defined pre-specified primary and secondary outcome measures, including how and when they were assessed

Does your paper address CONSORT subitem 6a? *

Copy and paste relevant sections from the manuscript (include quotes in quotation marks "like this" to indicate direct quotes from your manuscript), or elaborate on this item by providing additional information not in the ms, or briefly explain why the item is not applicable/relevant for your study

"Recruitment started in October 2020 and ended in May 2022."
"All measures were self-reported."

Details of primary and secondary outcome measures are described under Methods -> Procedure -> Outcome measures

6a-i) Online questionnaires: describe if they were validated for online use and apply CHERRIES items to describe how the questionnaires were designed/deployed

If outcomes were obtained through online questionnaires, describe if they were validated for online use and apply CHERRIES items to describe how the questionnaires were designed/deployed [9].

	1	2	3	4	5	
subitem not at all important	○	○	○	◉	○	essential

Auswahl löschen

Does your paper address subitem 6a-i?

Copy and paste relevant sections from manuscript text

Examples for reports according to CHERRIES:
"The whole study (including informed consent and gift card lottery pages) comprised 26 pages with 1 to 12 items on each page. Each segment or measure was presented on one or two separate pages, depending on its respective length. Some items were conditional for adaptive questioning (for example, when lifetime NSSI was denied, no further questions about NSSI were presented). Changes to the item responses could only be made while they have not been confirmed through a click on the “next”-button, which brought participants to the next page. There was no “back”-button."
"HTTP-cookies were used to assign individual user IDs to participants. For each session, new cookies were generated and utilized. Duplicate participation was therefore possible after the completion of each study session and was not registered by the system. In the five above-mentioned cases, duplicate IDs were mistakenly generated when participants tried to use the “back”-button of their web browser and re-started their participation."

6a-ii) Describe whether and how “use” (including intensity of use/dosage) was defined/measured/monitored

Describe whether and how “use” (including intensity of use/dosage) was defined/measured/monitored (logins, logfile analysis, etc.). Use/adoption metrics are important process outcomes that should be reported in any ehealth trial.

	1	2	3	4	5	
subitem not at all important	○	○	○	◉	○	essential

Auswahl löschen

Does your paper address subitem 6a-ii?

Copy and paste relevant sections from manuscript text

"We recorded page change timestamps. Participants whose timestamp data indicated that the video(s) have not been fully viewed (i.e. duration of stay < length of respective videos) were excluded from statistical analyses."

6a-iii) Describe whether, how, and when qualitative feedback from participants was obtained

Describe whether, how, and when qualitative feedback from participants was obtained (e.g., through emails, feedback forms, interviews, focus groups).

	1	2	3	4	5	
subitem not at all important	○	◉	○	○	○	essential

Auswahl löschen

Does your paper address subitem 6a-iii?

Copy and paste relevant sections from manuscript text

Meine Antwort

6b) Any changes to trial outcomes after the trial commenced, with reasons

Does your paper address CONSORT subitem 6b? *

Copy and paste relevant sections from the manuscript (include quotes in quotation marks "like this" to indicate direct quotes from your manuscript), or elaborate on this item by providing additional information not in the ms, or briefly explain why the item is not applicable/relevant for your study

Not applicable, no changes were made.

7a) How sample size was determined

NPT: When applicable, details of whether and how the clustering by care provides or centers was addressed

7a-i) Describe whether and how expected attrition was taken into account when calculating the sample size

Describe whether and how expected attrition was taken into account when calculating the sample size.

	1	2	3	4	5	
subitem not at all important	○	◉	○	○	○	essential

Auswahl löschen

Does your paper address subitem 7a-i?

Copy and paste relevant sections from manuscript title (include quotes in quotation marks "like this" to indicate direct quotes from your manuscript), or elaborate on this item by providing additional information not in the ms, or briefly explain why the item is not applicable/relevant for your study

Meine Antwort

7b) When applicable, explanation of any interim analyses and stopping guidelines

Does your paper address CONSORT subitem 7b? *

Copy and paste relevant sections from the manuscript (include quotes in quotation marks "like this" to indicate direct quotes from your manuscript), or elaborate on this item by providing additional information not in the ms, or briefly explain why the item is not applicable/relevant for your study

Not applicable. No interim analyses to inform data acquisition decisions or stopping guidelines were conducted. Preliminary study results were presented at conferences. However, they were solely presented to disseminate study information and were not intended to influence decisions on further data acquisition.

8a) Method used to generate the random allocation sequence

NPT: When applicable, how care providers were allocated to each trial group

Does your paper address CONSORT subitem 8a? *

Copy and paste relevant sections from the manuscript (include quotes in quotation marks "like this" to indicate direct quotes from your manuscript), or elaborate on this item by providing additional information not in the ms, or briefly explain why the item is not applicable/relevant for your study

"This anonymous, fully automated web-based parallel group exploratory RCT compared the effects of INT1 (psychoeducational intervention) and INT2 (positive consequences of help-seeking) against the control group (CG; no further video after case vignette) with regard to potential help-seeking, attitudes towards help-seeking, and stigma. The design comprised 15 conditions in total (5 MH problems x 3 interventional conditions). Randomization was stratified by gender and implemented with a permuted block design (block sizes: 15, 30)." "A randomization list with numbers representing the conditions has been generated and embedded in our ASMO-database [58] prior to recruitment." "R was also used to generate the random allocation sequence."

8b) Type of randomisation; details of any restriction (such as blocking and block size)

Does your paper address CONSORT subitem 8b? *

Copy and paste relevant sections from the manuscript (include quotes in quotation marks "like this" to indicate direct quotes from your manuscript), or elaborate on this item by providing additional information not in the ms, or briefly explain why the item is not applicable/relevant for your study

"This anonymous, fully automated web-based parallel group exploratory RCT compared the effects of INT1 (psychoeducational intervention) and INT2 (positive consequences of help-seeking) against the control group (CG; no further video after case vignette) with regard to potential help-seeking, attitudes towards help-seeking, and stigma. The design comprised 15 conditions in total (5 MH problems x 3 interventional conditions). Randomization was stratified by gender and implemented with a permuted block design (block sizes: 15, 30)."

9) Mechanism used to implement the random allocation sequence (such as sequentially numbered containers), describing any steps taken to conceal the sequence until interventions were assigned

Does your paper address CONSORT subitem 9? *

Copy and paste relevant sections from the manuscript (include quotes in quotation marks "like this" to indicate direct quotes from your manuscript), or elaborate on this item by providing additional information not in the ms, or briefly explain why the item is not applicable/relevant for your study

"A randomization list with numbers representing the conditions has been generated and embedded in our ASMO-database [58] prior to recruitment." "R was also used to generate the random allocation sequence."

10) Who generated the random allocation sequence, who enrolled participants, and who assigned participants to interventions

Does your paper address CONSORT subitem 10? *

Copy and paste relevant sections from the manuscript (include quotes in quotation marks "like this" to indicate direct quotes from your manuscript), or elaborate on this item by providing additional information not in the ms, or briefly explain why the item is not applicable/relevant for your study

Authors contributions: "MM generated the random allocation sequence. DL was responsible for study recruitment, which was supported by student assistants."

Abstract: "Out of N=2435 participants who commenced participation, a final sample of N=1394 participants (57%) aged 14 to 29 years with complete data and sufficient durations of stay on the video pages were randomized in a fully automated manner to one of the five MH problems and one of three conditions (control, INT1, INT2) in a permuted block design."

Methods: "This anonymous, fully automated web-based parallel group exploratory RCT compared the effects of INT1 (psychoeducational intervention) and INT2 (positive consequences of help-seeking) against the control group (CG; no further video after case vignette) with regard to potential help-seeking, attitudes towards help-seeking, and stigma."

11a) If done, who was blinded after assignment to interventions (for example, participants, care providers, those assessing outcomes) and how

NPT: Whether or not administering co-interventions were blinded to group assignment

11a-i) Specify who was blinded, and who wasn't

Specify who was blinded, and who wasn't. Usually, in web-based trials it is not possible to blind the participants [1, 3] (this should be clearly acknowledged), but it may be possible to blind outcome assessors, those doing data analysis or those administering co-interventions (if any).

	1	2	3	4	5	
subitem not at all important	○	○	◉	○	○	essential

Auswahl löschen

Does your paper address subitem 11a-i? *

Copy and paste relevant sections from the manuscript (include quotes in quotation marks "like this" to indicate direct quotes from your manuscript), or elaborate on this item by providing additional information not in the ms, or briefly explain why the item is not applicable/relevant for your study

"Since the aim of the conditions was to provide information about a specific MH problem, blinding of participants after assignment to interventions did not occur."
"Authors involved in data analysis and interpretation were not blinded with respect to the assigned experimental conditions."
"Due to anonymous participation and automated randomization, researchers were unable to assign specific conditions to individuals. However, two of the authors were able to view the randomization list."

11a-ii) Discuss e.g., whether participants knew which intervention was the "intervention of interest" and which one was the "comparator"

Informed consent procedures (4a-ii) can create biases and certain expectations - discuss e.g., whether participants knew which intervention was the "intervention of interest" and which one was the "comparator".

	1	2	3	4	5	
subitem not at all important	○	○	○	◉	○	essential

Auswahl löschen

Does your paper address subitem 11a-ii?

Copy and paste relevant sections from the manuscript (include quotes in quotation marks "like this" to indicate direct quotes from your manuscript), or elaborate on this item by providing additional information not in the ms, or briefly explain why the item is not applicable/relevant for your study

"Participants were informed that they would be randomly assigned to one out of five health problems and one out of three video versions. They were not informed about the specific health issues or the conditions' details prior to participation."

11b) If relevant, description of the similarity of interventions

(this item is usually not relevant for ehealth trials as it refers to similarity of a placebo or sham intervention to a active medication/intervention)

Does your paper address CONSORT subitem 11b? *

Copy and paste relevant sections from the manuscript (include quotes in quotation marks "like this" to indicate direct quotes from your manuscript), or elaborate on this item by providing additional information not in the ms, or briefly explain why the item is not applicable/relevant for your study

Not applicable. Sham or placebo interventions were not part of this study.

12a) Statistical methods used to compare groups for primary and secondary outcomes

NPT: When applicable, details of whether and how the clustering by care providers or centers was addressed

Does your paper address CONSORT subitem 12a? *

Copy and paste relevant sections from the manuscript (include quotes in quotation marks "like this" to indicate direct quotes from your manuscript), or elaborate on this item by providing additional information not in the ms, or briefly explain why the item is not applicable/relevant for your study

"Intervention effects on potential professional help-seeking (primary outcome) and secondary outcomes in the total sample (i.e., across all MH problems and across participants with and without actual help-seeking, as reported in the screening) were analyzed via analyses of covariance (ANCOVAs) at an alpha level of P<.05. In addition to the intervention group, the models included age as a covariate, the participants' actual help-seeking (fixed effects), and the 5 MH problems (random effects) as control variables."
"In case of significant (P<.05) and trend ANCOVA effects, pairwise group-comparisons were conducted using t-tests. All tests were two-sided with an alpha level of 5%. Mean differences (MDadj.) adjusted for covariates are reported in the results section below."

12a-i) Imputation techniques to deal with attrition / missing values

Imputation techniques to deal with attrition / missing values: Not all participants will use the intervention/comparator as intended and attrition is typically high in ehealth trials. Specify how participants who did not use the application or dropped out from the trial were treated in the statistical analysis (a complete case analysis is strongly discouraged, and simple imputation techniques such as LOCF may also be problematic [4]).

	1	2	3	4	5	
subitem not at all important	○	○	○	◉	○	essential

Auswahl löschen

Does your paper address subitem 12a-i? *

Copy and paste relevant sections from the manuscript (include quotes in quotation marks "like this" to indicate direct quotes from your manuscript), or elaborate on this item by providing additional information not in the ms, or briefly explain why the item is not applicable/relevant for your study

"Out of the 2208 participants who were randomized to one of the 15 conditions, n=472 were excluded because their time spent on the video pages fell below the durations of the videos they were assigned to, indicating that they did not view the entire video(s). Out of the remaining 1736 participants, n=342 were excluded due to incomplete data, i.e. they did not complete all of the relevant scales the study entailed (beginning with informed consent up to and including the last acceptability item). Our final sample consisted of N=1394 youths aged 14 to 29 years (M=20.97, SD=3.67)."

12b) Methods for additional analyses, such as subgroup analyses and adjusted analyses

Does your paper address CONSORT subitem 12b? *

Copy and paste relevant sections from the manuscript (include quotes in quotation marks "like this" to indicate direct quotes from your manuscript), or elaborate on this item by providing additional information not in the ms, or briefly explain why the item is not applicable/relevant for your study

"Subgroup ANCOVAs were conducted for each of the five MH problems separately. Here, the respective screening scores (GAD-7, PHQ-9, WCS, number of NSSI events during the last year, AUDIT-C) were included as additional covariates. Subgroup analyses were further conducted for cases with and without actual help-seeking in the total sample and within each of the five MH issue groups.
In case of significant (P<.05) and trend ANCOVA effects, pairwise group-comparisons were conducted using t-tests. All tests were two-sided with an alpha level of 5%. Mean differences (MDadj.) adjusted for covariates are reported in the results section below."

X26) REB/IRB Approval and Ethical Considerations [recommended as subheading under "Methods"] (not a CONSORT item)

X26-i) Comment on ethics committee approval

	1	2	3	4	5	
subitem not at all important	○	○	○	●	○	essential

Auswahl löschen

Does your paper address subitem X26-i?

Copy and paste relevant sections from the manuscript (include quotes in quotation marks "like this" to indicate direct quotes from your manuscript), or elaborate on this item by providing additional information not in the ms, or briefly explain why the item is not applicable/relevant for your study

"Ethical approval was obtained from the Ethics Committee of the Heidelberg Medical Faculty (S-378/2020)."
"Ethical approval was obtained from the Ethics Committee I of the Heidelberg Medical Faculty on 27th of July, 2020, protocol number S-378/2020."

x26-ii) Outline informed consent procedures

Outline informed consent procedures e.g., if consent was obtained offline or online (how? Checkbox, etc.?), and what information was provided (see 4a-ii). See [6] for some items to be included in informed consent documents.

	1	2	3	4	5	
subitem not at all important	○	○	●	○	○	essential

Auswahl löschen

Does your paper address subitem X26-ii?

Copy and paste relevant sections from the manuscript (include quotes in quotation marks "like this" to indicate direct quotes from your manuscript), or elaborate on this item by providing additional information not in the ms, or briefly explain why the item is not applicable/relevant for your study

"Prior to their participation, youths received detailed information about the aims, scope, procedures, data processing, and data storage of the study on the website. Participants were informed that they would be randomly assigned to one out of five health problems and one out of three video versions. They were not informed about the specific health issues or the conditions' details prior to participation. Since the aim of the conditions was to provide information about a specific MH problem, blinding of participants after assignment to interventions did not occur. Only participants who provided informed consent through an online-checkbox were eligible for participation. After study completion, participants were debriefed in writing about the objectives on the study website. The debriefing form also included contact information of formal help services."

X26-iii) Safety and security procedures

Safety and security procedures, incl. privacy considerations, and any steps taken to reduce the likelihood or detection of harm (e.g., education and training, availability of a hotline)

	1	2	3	4	5	
subitem not at all important	○	◉	○	○	○	essential

Auswahl löschen

Does your paper address subitem X26-iii?

Copy and paste relevant sections from the manuscript (include quotes in quotation marks "like this" to indicate direct quotes from your manuscript), or elaborate on this item by providing additional information not in the ms, or briefly explain why the item is not applicable/relevant for your study

Meine Antwort

RESULTS

13a) For each group, the numbers of participants who were randomly assigned, received intended treatment, and were analysed for the primary outcome

NPT: The number of care providers or centers performing the intervention in each group and the number of patients treated by each care provider in each center

Does your paper address CONSORT subitem 13a? *

Copy and paste relevant sections from the manuscript (include quotes in quotation marks "like this" to indicate direct quotes from your manuscript), or elaborate on this item by providing additional information not in the ms, or briefly explain why the item is not applicable/relevant for your study

See Table 2. ANCOVA results and pairwise comparisons for primary outcomes (total sample).
See Figure 1. Study design and sample sizes.

13b) For each group, losses and exclusions after randomisation, together with reasons

Does your paper address CONSORT subitem 13b? (NOTE: Preferably, this is shown in a CONSORT flow diagram) *

Copy and paste relevant sections from the manuscript (include quotes in quotation marks "like this" to indicate direct quotes from your manuscript), or elaborate on this item by providing additional information not in the ms, or briefly explain why the item is not applicable/relevant for your study

See Figure 1. Study design and sample sizes.

13b-i) Attrition diagram

Strongly recommended: An attrition diagram (e.g., proportion of participants still logging in or using the intervention/comparator in each group plotted over time, similar to a survival curve) or other figures or tables demonstrating usage/dose/engagement.

	1	2	3	4	5	
subitem not at all important	○	○	◉	○	○	essential

Auswahl löschen

Does your paper address subitem 13b-i?

Copy and paste relevant sections from the manuscript or cite the figure number if applicable (include quotes in quotation marks "like this" to indicate direct quotes from your manuscript), or elaborate on this item by providing additional information not in the ms, or briefly explain why the item is not applicable/relevant for your study

Figure 1 comprises excluded cases due to insufficient engagement (i.e. durations of stay on video pages)

14a) Dates defining the periods of recruitment and follow-up

Does your paper address CONSORT subitem 14a? *

Copy and paste relevant sections from the manuscript (include quotes in quotation marks "like this" to indicate direct quotes from your manuscript), or elaborate on this item by providing additional information not in the ms, or briefly explain why the item is not applicable/relevant for your study

"Recruitment started in October 2020 and ended in May 2022." (no follow-up)

14a-i) Indicate if critical “secular events” fell into the study period

Indicate if critical “secular events” fell into the study period, e.g., significant changes in Internet resources available or “changes in computer hardware or Internet delivery resources”

	1	2	3	4	5	
subitem not at all important	○	◉	○	○	○	essential

Auswahl löschen

Does your paper address subitem 14a-i?

Copy and paste relevant sections from the manuscript (include quotes in quotation marks "like this" to indicate direct quotes from your manuscript), or elaborate on this item by providing additional information not in the ms, or briefly explain why the item is not applicable/relevant for your study

Meine Antwort

14b) Why the trial ended or was stopped (early)

Does your paper address CONSORT subitem 14b? *

Copy and paste relevant sections from the manuscript (include quotes in quotation marks "like this" to indicate direct quotes from your manuscript), or elaborate on this item by providing additional information not in the ms, or briefly explain why the item is not applicable/relevant for your study

Not applicable, recruitment ended when enough data were collected.

15) A table showing baseline demographic and clinical characteristics for each group

NPT: When applicable, a description of care providers (case volume, qualification, expertise, etc.) and centers (volume) in each group

Does your paper address CONSORT subitem 15? *

Copy and paste relevant sections from the manuscript (include quotes in quotation marks "like this" to indicate direct quotes from your manuscript), or elaborate on this item by providing additional information not in the ms, or briefly explain why the item is not applicable/relevant for your study

Table 1. Sociodemographic characteristics and screening results contains demographic and clinical characteristics for the total sample. Tables with sociodemographic information in each group were not included to limit the number of tables and to ensure clarity of the manuscript.

15-i) Report demographics associated with digital divide issues

In ehealth trials it is particularly important to report demographics associated with digital divide issues, such as age, education, gender, social-economic status, computer/Internet/ehealth literacy of the participants, if known.

	1	2	3	4	5	
subitem not at all important	○	○	◉	○	○	essential

Auswahl löschen

Does your paper address subitem 15-i? *

Copy and paste relevant sections from the manuscript (include quotes in quotation marks "like this" to indicate direct quotes from your manuscript), or elaborate on this item by providing additional information not in the ms, or briefly explain why the item is not applicable/relevant for your study

Table 1. Sociodemographic characteristics and screening results.

16) For each group, number of participants (denominator) included in each analysis and whether the analysis was by original assigned groups

16-i) Report multiple “denominators” and provide definitions

Report multiple “denominators” and provide definitions: Report N’s (and effect sizes) “across a range of study participation [and use] thresholds” [1], e.g., N exposed, N consented, N used more than x times, N used more than y weeks, N participants “used” the intervention/comparator at specific pre-defined time points of interest (in absolute and relative numbers per group). Always clearly define “use” of the intervention.

	1	2	3	4	5	
subitem not at all important	○	○	○	○	◉	essential

Auswahl löschen

Does your paper address subitem 16-i? *

Copy and paste relevant sections from the manuscript (include quotes in quotation marks "like this" to indicate direct quotes from your manuscript), or elaborate on this item by providing additional information not in the ms, or briefly explain why the item is not applicable/relevant for your study

Table 2. ANCOVA results and pairwise comparisons for primary outcomes (total sample).
Appendices 1-3

16-ii) Primary analysis should be intent-to-treat

Primary analysis should be intent-to-treat, secondary analyses could include comparing only “users”, with the appropriate caveats that this is no longer a randomized sample (see 18-i).

	1	2	3	4	5	
subitem not at all important	○	◉	○	○	○	essential

Auswahl löschen

Does your paper address subitem 16-ii?

Copy and paste relevant sections from the manuscript (include quotes in quotation marks "like this" to indicate direct quotes from your manuscript), or elaborate on this item by providing additional information not in the ms, or briefly explain why the item is not applicable/relevant for your study

Meine Antwort

17a) For each primary and secondary outcome, results for each group, and the estimated effect size and its precision (such as 95% confidence interval)

Does your paper address CONSORT subitem 17a? *

Copy and paste relevant sections from the manuscript (include quotes in quotation marks "like this" to indicate direct quotes from your manuscript), or elaborate on this item by providing additional information not in the ms, or briefly explain why the item is not applicable/relevant for your study

Table 2. ANCOVA results and pairwise comparisons for primary outcomes (total sample).
Appendices 1-3
Figure 2. ANCOVA results (overview).
Example: "Across all MH problems, no statistically significant group main effect was found on potential professional help-seeking ($F(2,1385)=.99$, $P=.37$) (Table 2)."

17a-i) Presentation of process outcomes such as metrics of use and intensity of use

In addition to primary/secondary (clinical) outcomes, the presentation of process outcomes such as metrics of use and intensity of use (dose, exposure) and their operational definitions is critical. This does not only refer to metrics of attrition (13-b) (often a binary variable), but also to more continuous exposure metrics such as "average session length". These must be accompanied by a technical description how a metric like a "session" is defined (e.g., timeout after idle time) [1] (report under item 6a).

	1	2	3	4	5	
subitem not at all important	○	○	◉	○	○	essential

Auswahl löschen

Does your paper address subitem 17a-i?

Copy and paste relevant sections from the manuscript (include quotes in quotation marks "like this" to indicate direct quotes from your manuscript), or elaborate on this item by providing additional information not in the ms, or briefly explain why the item is not applicable/relevant for your study

Meine Antwort

17b) For binary outcomes, presentation of both absolute and relative effect sizes is recommended

Does your paper address CONSORT subitem 17b? *

Copy and paste relevant sections from the manuscript (include quotes in quotation marks "like this" to indicate direct quotes from your manuscript), or elaborate on this item by providing additional information not in the ms, or briefly explain why the item is not applicable/relevant for your study

Not applicable, no binary outcomes.

18) Results of any other analyses performed, including subgroup analyses and adjusted analyses, distinguishing pre-specified from exploratory

Does your paper address CONSORT subitem 18? *

Copy and paste relevant sections from the manuscript (include quotes in quotation marks "like this" to indicate direct quotes from your manuscript), or elaborate on this item by providing additional information not in the ms, or briefly explain why the item is not applicable/relevant for your study

Appendices 1-3
Figure 2. ANCOVA results (overview).

18-i) Subgroup analysis of comparing only users

A subgroup analysis of comparing only users is not uncommon in ehealth trials, but if done, it must be stressed that this is a self-selected sample and no longer an unbiased sample from a randomized trial (see 16-iii).

	1	2	3	4	5	
subitem not at all important	○	◉	○	○	○	essential

Auswahl löschen

Does your paper address subitem 18-i?

Copy and paste relevant sections from the manuscript (include quotes in quotation marks "like this" to indicate direct quotes from your manuscript), or elaborate on this item by providing additional information not in the ms, or briefly explain why the item is not applicable/relevant for your study

Meine Antwort

19) All important harms or unintended effects in each group

(for specific guidance see CONSORT for harms)

Does your paper address CONSORT subitem 19? *

Copy and paste relevant sections from the manuscript (include quotes in quotation marks "like this" to indicate direct quotes from your manuscript), or elaborate on this item by providing additional information not in the ms, or briefly explain why the item is not applicable/relevant for your study

Not applicable. Due to the aim (micro-intervention with randomized content) and target group (general youth sample) harms were not expected and not explicitly measured. However, the debriefing form included contact information of formal help services in case participants wished to seek support:
"After study completion, participants were debriefed in writing about the objectives on the study website. The debriefing form also included contact information of formal help services."

19-i) Include privacy breaches, technical problems

Include privacy breaches, technical problems. This does not only include physical “harm” to participants, but also incidents such as perceived or real privacy breaches [1], technical problems, and other unexpected/unintended incidents. “Unintended effects” also includes unintended positive effects [2].

	1	2	3	4	5	
subitem not at all important	○	○	◉	○	○	essential

Auswahl löschen

Does your paper address subitem 19-i?

Copy and paste relevant sections from the manuscript (include quotes in quotation marks "like this" to indicate direct quotes from your manuscript), or elaborate on this item by providing additional information not in the ms, or briefly explain why the item is not applicable/relevant for your study

Meine Antwort

19-ii) Include qualitative feedback from participants or observations from staff/researchers

Include qualitative feedback from participants or observations from staff/researchers, if available, on strengths and shortcomings of the application, especially if they point to unintended/unexpected effects or uses. This includes (if available) reasons for why people did or did not use the application as intended by the developers.

	1	2	3	4	5	
subitem not at all important	○	○	◉	○	○	essential

Auswahl löschen

Does your paper address subitem 19-ii?

Copy and paste relevant sections from the manuscript (include quotes in quotation marks "like this" to indicate direct quotes from your manuscript), or elaborate on this item by providing additional information not in the ms, or briefly explain why the item is not applicable/relevant for your study

Meine Antwort

DISCUSSION

22) Interpretation consistent with results, balancing benefits and harms, and considering other relevant evidence

NPT: In addition, take into account the choice of the comparator, lack of or partial blinding, and unequal expertise of care providers or centers in each group

22-i) Restate study questions and summarize the answers suggested by the data, starting with primary outcomes and process outcomes (use)

Restate study questions and summarize the answers suggested by the data, starting with primary outcomes and process outcomes (use).

	1	2	3	4	5	
subitem not at all important	○	○	○	●	○	essential

Auswahl löschen

Does your paper address subitem 22-i? *

Copy and paste relevant sections from the manuscript (include quotes in quotation marks "like this" to indicate direct quotes from your manuscript), or elaborate on this item by providing additional information not in the ms, or briefly explain why the item is not applicable/relevant for your study

Example: "This study developed and tested the short-term effectiveness of two brief video-based strategies targeted at adolescents and young adults (14 to 29 years), aiming to foster potential professional help-seeking (main outcome) and related attitudes for five MH problems. In the total sample, we did not find effects of either INT1 (psychoeducation) or INT2 (positive outcome expectancies) on our primary outcome. However, significant group effects were found with respect to potential informal help-seeking, stigma towards others, and indifference to stigma in the total sample."

22-ii) Highlight unanswered new questions, suggest future research

Highlight unanswered new questions, suggest future research.

	1	2	3	4	5	
subitem not at all important	○	○	○	○	◉	essential

Auswahl löschen

Does your paper address subitem 22-ii?

Copy and paste relevant sections from the manuscript (include quotes in quotation marks "like this" to indicate direct quotes from your manuscript), or elaborate on this item by providing additional information not in the ms, or briefly explain why the item is not applicable/relevant for your study

Example:
"Future research should focus on improvements in effectiveness of micro-interventions. One approach could be the investigation of interventional framing. In a Japanese study on depression, loss-framed messages (i.e. emphasizing negative consequences of refraining from help-seeking) had a greater impact on help-seeking intentions than gain-framed (positive consequences of help-seeking) or neutral messages (e.g. prevalence rates) as well as unformatted, plain text messages in middle-aged adults [90]. It might thus be interesting to conduct future studies on the effects of video-based micro-interventions with differently framed messaging, since the videos in this study emphasized potential gains of help-seeking rather than potential losses of help-seeking restraint."

20) Trial limitations, addressing sources of potential bias, imprecision, and, if relevant, multiplicity of analyses

20-i) Typical limitations in ehealth trials

Typical limitations in ehealth trials: Participants in ehealth trials are rarely blinded. Ehealth trials often look at a multiplicity of outcomes, increasing risk for a Type I error. Discuss biases due to non-use of the intervention/usability issues, biases through informed consent procedures, unexpected events.

	1	2	3	4	5	
subitem not at all important	○	○	○	○	◉	essential

Auswahl löschen

Does your paper address subitem 20-i? *

Copy and paste relevant sections from the manuscript (include quotes in quotation marks "like this" to indicate direct quotes from your manuscript), or elaborate on this item by providing additional information not in the ms, or briefly explain why the item is not applicable/relevant for your study

Examples (Limitations):

"One limitation of this study lies within the sole investigation of effects on hypothetical intentions and attitudes instead of actual help-seeking behavior. While intentions are substantially associated with behavior and provide valuable insights, they do not translate directly into behavioral change [49]. Moreover, only short-term effects were investigated."

"We also included trend effects in our overview (Figure 2), which should be interpreted with caution. However, these findings might be useful to inform the planning of subsequent research in this field."

"Lastly, we did not include a comprehension check to assess participants' understanding of and engagement with the content presented in the videos. While we accounted for the time participants spent on the video pages and only included participants with sufficient durations of stay in the final analyses, they may not have fully comprehended or attended to the video material. The substantial number of excluded participants who completed the study without meeting the time threshold (n=472) underlines this potential issue. Future studies should thus address this limitation by including comprehension checks to improve the robustness of findings."

21) Generalisability (external validity, applicability) of the trial findings

NPT: External validity of the trial findings according to the intervention, comparators, patients, and care providers or centers involved in the trial

21-i) Generalizability to other populations

Generalizability to other populations: In particular, discuss generalizability to a general Internet population, outside of a RCT setting, and general patient population, including applicability of the study results for other organizations

	1	2	3	4	5	
subitem not at all important	○	○	○	◉	○	essential

Auswahl löschen

Does your paper address subitem 21-i?

Copy and paste relevant sections from the manuscript (include quotes in quotation marks "like this" to indicate direct quotes from your manuscript), or elaborate on this item by providing additional information not in the ms, or briefly explain why the item is not applicable/relevant for your study

"Furthermore, our sample demonstrated, on average, a high level of education, very little public stigma, a pronounced willingness to seek help, and a high rate of actual professional help-seeking (approximately 45%), which limits the generalizability of our findings. More than 90% of our sample knew someone with MH problems. While we were careful not to recruit MH experts, such as university students of medicine and psychology, youths with a personal interest in MH related topics seemed to have been more inclined to participate. Related to this, we aimed for a community youth sample rather than a clinical sample. The scenarios which our items referred to were hypothetical and did not necessarily reflect participants' own experiences due to the random assignment to one of the five MH problems. A similar approach with targeted interventions according to youths' actual MH status and more individualized elements with regard to gender-related [91] and cultural [92, 93] aspects could be promising in future research. Upcoming studies should thus strive to align their research objectives more closely with the characteristics and needs of the selected target groups."

21-ii) Discuss if there were elements in the RCT that would be different in a routine application setting

Discuss if there were elements in the RCT that would be different in a routine application setting (e.g., prompts/reminders, more human involvement, training sessions or other co-interventions) and what impact the omission of these elements could have on use, adoption, or outcomes if the intervention is applied outside of a RCT setting.

	1	2	3	4	5	
subitem not at all important	○	○	◉	○	○	essential

Auswahl löschen

Does your paper address subitem 21-ii?

Copy and paste relevant sections from the manuscript (include quotes in quotation marks "like this" to indicate direct quotes from your manuscript), or elaborate on this item by providing additional information not in the ms, or briefly explain why the item is not applicable/relevant for your study

Meine Antwort

OTHER INFORMATION

23) Registration number and name of trial registry

Does your paper address CONSORT subitem 23? *

Copy and paste relevant sections from the manuscript (include quotes in quotation marks "like this" to indicate direct quotes from your manuscript), or elaborate on this item by providing additional information not in the ms, or briefly explain why the item is not applicable/relevant for your study

"Trial registration: This study has been registered at the German Clinical Trials Register (www.drks.de) on September 23rd, 2020: https://drks.de/search/de/trial/DRKS00023110 #DRKS00023110."

24) Where the full trial protocol can be accessed, if available

Does your paper address CONSORT subitem 24? *

Cite a Multimedia Appendix, other reference, or copy and paste relevant sections from the manuscript (include quotes in quotation marks "like this" to indicate direct quotes from your manuscript), or elaborate on this item by providing additional information not in the ms, or briefly explain why the item is not applicable/relevant for your study

"Trial registration: This study has been registered at the German Clinical Trials Register (www.drks.de) on September 23rd, 2020: https://drks.de/search/de/trial/DRKS00023110 #DRKS00023110."

25) Sources of funding and other support (such as supply of drugs), role of funders

Does your paper address CONSORT subitem 25? *

Copy and paste relevant sections from the manuscript (include quotes in quotation marks "like this" to indicate direct quotes from your manuscript), or elaborate on this item by providing additional information not in the ms, or briefly explain why the item is not applicable/relevant for your study

"Funding
This study was funded by the German Federal Ministry of Education and Research (BMBF) (funding identifier: 01GL1904). The BMBF had no influence on the design of the study and was not involved in data collection, analysis and interpretation, or the writing of manuscripts."

X27) Conflicts of Interest (not a CONSORT item)

X27-i) State the relation of the study team towards the system being evaluated

In addition to the usual declaration of interests (financial or otherwise), also state the relation of the study team towards the system being evaluated, i.e., state if the authors/evaluators are distinct from or identical with the developers/sponsors of the intervention.

	1	2	3	4	5	
subitem not at all important	○	○	◉	○	○	essential

Auswahl löschen

Does your paper address subitem X27-i?

Copy and paste relevant sections from the manuscript (include quotes in quotation marks "like this" to indicate direct quotes from your manuscript), or elaborate on this item by providing additional information not in the ms, or briefly explain why the item is not applicable/relevant for your study

Meine Antwort

About the CONSORT EHEALTH checklist

As a result of using this checklist, did you make changes in your manuscript? *

- ○ yes, major changes
- ◉ yes, minor changes
- ○ no

What were the most important changes you made as a result of using this checklist?

Meine Antwort

How much time did you spend on going through the checklist INCLUDING making changes in your manuscript *

Two to three work days (several hours, but I do not recall how many exactly)

As a result of using this checklist, do you think your manuscript has improved? *

- ○ yes
- ○ no
- ◉ Sonstiges: It sure has improved in some aspects; however, our study is more

Would you like to become involved in the CONSORT EHEALTH group?

This would involve for example becoming involved in participating in a workshop and writing an "Explanation and Elaboration" document

- ○ yes
- ◉ no
- ○ Sonstiges:

Auswahl löschen

Any other comments or questions on CONSORT EHEALTH

Meine Antwort

STOP - Save this form as PDF before you click submit

To generate a record that you filled in this form, we recommend to generate a PDF of this page (on a Mac, simply select "print" and then select "print as PDF") before you submit it.

When you submit your (revised) paper to JMIR, please upload the PDF as supplementary file.

Don't worry if some text in the textboxes is cut off, as we still have the complete information in our database. Thank you!

Final step: Click submit !

Click submit so we have your answers in our database!

Senden | Alle Eingaben löschen

Geben Sie niemals Passwörter über Google Formulare weiter.

Dieser Inhalt wurde nicht von Google erstellt und wird von Google auch nicht unterstützt. Missbrauch melden - Nutzungsbedingungen - Datenschutzerklärung

Google Formulare
